# What Does Carotenoid-Dependent Coloration Tell? Plasma Carotenoid Level Signals Immunocompetence and Oxidative Stress State in Birds–A Meta-Analysis

**DOI:** 10.1371/journal.pone.0043088

**Published:** 2012-08-14

**Authors:** Mirre J. P. Simons, Alan A. Cohen, Simon Verhulst

**Affiliations:** 1 Behavioural Biology, University of Groningen, Groningen, The Netherlands; 2 Department of Family Medicine, University of Sherbrooke, Sherbrooke, Quebec, Canada; Utrecht University, The Netherlands

## Abstract

Mechanisms maintaining honesty of sexual signals are far from resolved, limiting our understanding of sexual selection and potential important parts of physiology. Carotenoid pigmented visual signals are among the most extensively studied sexual displays, but evidence regarding hypotheses on how carotenoids ensure signal honesty is mixed. Using a phylogenetically controlled meta-analysis of 357 effect sizes across 88 different species of birds, we tested two prominent hypotheses in the field: that carotenoid-dependent coloration signals i) immunocompetence and/or ii) oxidative stress state. Separate meta-analyses were performed for the relationships of trait coloration and circulating carotenoid level with different measures of immunocompetence and oxidative stress state. For immunocompetence we find that carotenoid levels (*r* = 0.20) and trait color intensity (*r* = 0.17) are significantly positively related to PHA response. Additionally we find that carotenoids are significantly positively related to antioxidant capacity (*r* = 0.10), but not significantly related to oxidative damage (*r* = −0.02). Thus our analyses provide support for both hypotheses, in that at least for some aspects of immunity and oxidative stress state the predicted correlations were found. Furthermore, we tested for differences in effect size between experimental and observational studies; a larger effect in observational studies would indicate that co-variation might not be causal. However, we detected no significant difference, suggesting that the relationships we found are causal. The overall effect sizes we report are modest and we discuss potential factors contributing to this, including differences between species. We suggest complementary mechanisms maintaining honesty rather than the involvement of carotenoids in immune function and oxidative stress and suggest experiments on how to test these.

## Introduction

Mate choice for highly ornamented partners is common in the animal kingdom [1]. These ornaments are usually considered to have evolved through Fisherian runaway selection processes [2] or sensory drive [3], and can evolve into honest signals of phenotypic quality [4], [5]. Small preferences at the population level can rapidly select for increased chooser preference and for increased ornamentation of the chosen sex, given that attractive offspring result from sex with attractive mates. Associated costs of ornaments can limit their further elaboration [2]. For instance, if resources required for the development of the ornament are limited this prevents further elaboration. Variation in the ornament can now honestly signal genetic and phenotypic variation in the ability to acquire and/or maintain these resources [4]–[6]. Therefore choosing mates with these costly signals yields indirect genetic benefits, siring offspring that will be attractive and of high quality. It can also yield direct benefits if the costs of the ornament reflect or are directly related to resources that underlie variation in reproductive performance [7]. Theoretically, sexual ornaments are thus predicted to reliably signal phenotypic quality. However, empirical evidence of costs is scarce [6], [8]. The handicap principle [4], [5] states that strategic investment into sexual signals, at the expense of some cost, maintains signal honesty. Not all honest signals require handicapping, but can also be maintained via a diversity of other mechanisms, (reviewed in [8], e.g. social punishment of cheaters). The operating honesty maintenance mechanism and its evolution can only be understood by identifying the fitness costs of sexual signals. Additionally, honest handicap signals which feature in mate choice are predicted to be closely linked to important physiological processes within the animal [9], given that this provides most signaling value and makes it difficult to avoid costs (i.e. cheat). The study of sexual signaling will thus likely yield both insights into its evolution and also into important physiological trade-offs.

Carotenoid dependent sexual traits have received considerable attention with respect to the mechanisms that could maintain their honesty [10]–[15]. Mate choice for more elaborate carotenoid dependent traits has been described in multiple species (e.g. [16]–[21]). Carotenoids cannot be synthesized *de novo* by vertebrates making them a scarce commodity [12]. Indeed supplementation with carotenoids increases redness of sexual traits (e.g. [22]–[25]).

Carotenoids have multiple functions, including the chemical potential to act as antioxidants [26]. However the significance of their role as antioxidant *in vivo* has been questioned [26], [27]. This was corroborated by an earlier meta-analysis in birds which reported no association between carotenoid level and oxidative stress state [28]. Antioxidants prevent damage by free radicals, e.g. reactive oxygen species, to crucial parts of the cell, such as DNA [29]. When antioxidant systems do not adequately quench free radicals, oxidative damage to cell components is increased, which is termed oxidative stress [29], [30]. In life history theory, oxidative stress has been hypothesized to shape lifespan and reproductive investment [31]. Possibly ignoring the debatable *in vivo* antioxidant potential of carotenoids [26], [27], redness of carotenoid dependent ornaments has been hypothesized to signal oxidative stress state of individuals [14], [15], [32]. The supplementation of non-carotenoid antioxidants generally increases carotenoid dependent sexual coloration [33]–[35], but see [25], suggesting that oxidative stress is involved in the determination of carotenoid-dependent coloration.

If redness of sexual traits reliably signals oxidative stress state, mate choice for these traits should yield direct and/or indirect fitness benefits. The precise mechanism through which carotenoid availability is honestly signaling oxidative stress state might be more complicated than carotenoids serving a substantial antioxidant role *in vivo*, but this does not mean that it is not doing just that. Carotenoid levels may function as indicators of oxidative damage, without contributing substantially to the antioxidant barrier, but indicating damage that is not adequately quenched by other antioxidants [27], [36]. The reason why these other antioxidants are not used in pigmentation of sexual signals, signaling antioxidant capacity more directly, may simply be because these antioxidants do not absorb light in the way that carotenoids do [27].

Another mechanism by which carotenoid levels may honestly signal aspects of condition is their role in supporting immune function. The immune system is one of the main contributors to total free radical production in vertebrates, and measures of oxidative stress state increase when birds are faced with an immune challenge [37]. The immune system itself is however also sensitive to oxidative stress compromising the integrity of immune cells, especially so because their plasma membranes contain large amounts of polyunsaturated fatty acids [38]. Immunosenescence is also attributed to increased oxidative stress, and has been shown to be reversed by antioxidant treatment [38], [39]. Carotenoids may therefore improve immune system functioning via their (debated) antioxidant function [40]–[42]. Alternatively carotenoids may also improve immune functioning via retinoids, which are derived from carotenoids [43], [44]. Retinoids also serve a wide range of other physiological roles involved in tissue repair and gene regulation [27].

**Figure 1:**
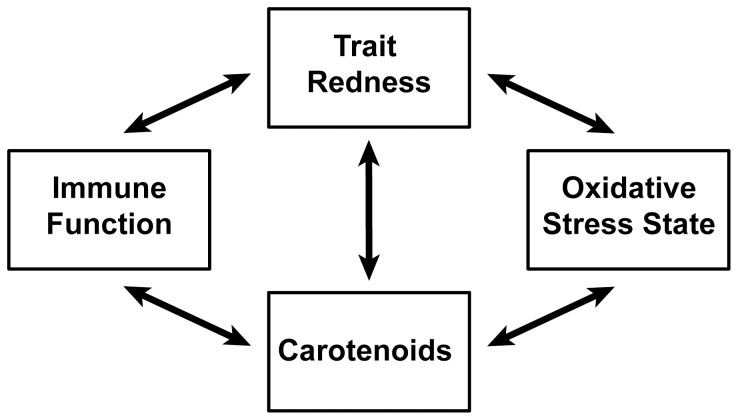
The five connections we investigated between, trait redness, carotenoids, immune function and oxidative stress state.

The aim of this study was to examine whether the honesty of carotenoid-dependent signals is maintained via the antioxidant and/or immune function action of carotenoids. To this end we carried out meta-analyses. In meta-analysis standardized metrics of multiple study outcomes, effect sizes (ESs) [45], [46], are combined to test hypotheses across studies [47], [48]. We focused on one class in the animal kingdom, birds, in which carotenoid-dependent signaling is both prevalent [49]–[51] and mechanistically studied [49]. We summarized five phenotypic relationships: circulating carotenoid levels with trait redness, immune function and oxidative stress state; and trait redness with immune function and oxidative stress state (figure 1). The relationships with trait redness represent signaling value, i.e. the information that can be obtained by a choosing individual regarding the physiological state of the signaler. The relationships with carotenoid levels represent the hypothesized mechanisms maintaining signal honesty.

## Methods and Approach

### Literature Search

Literature searches were conducted using Google Scholar, with our last search dating to January 2012. We used wide search terms (PRISMA S1, S2) which resulted in the screening of the maximum of 1000 hits Google Scholar provides. Additionally we screened the references of the articles we viewed full text. The details on the number of studies screened for eligibility is therefore a minimal estimate, given that cross-referencing was also used. Our search is described according to the PRISMA [52] flowchart (PRISMA S1). In our field of research no standard reviewing protocols exist that we could use.

Our exclusion criteria were as follows: i) Animals other than birds were studied ii) Necessary information to calculate effect size was not reported and authors did not respond to requests for this information. Authors were always contacted when information necessary to calculate relevant effect sizes was not reported. iii) An immune challenge or oxidative stress challenge was given after which carotenoid levels or sexual coloration were assessed. Our focus here is whether carotenoid levels or carotenoid-dependent coloration predict oxidative stress parameters or immune response. The question of whether challenges reduce carotenoid levels or redness of sexual coloration is relevant [53], [54], and this mechanism may in part or fully underlie between individual variation in sexual coloration. However, the effects of experimentally induced immune or oxidative stress cannot be directly scaled to natural variation or direct manipulation of carotenoid levels and may involve different trade-offs and hence we excluded such studies. iv) When carotenoid supplementation was applied experimentally, but data on natural variation in circulating carotenoid levels or coloration were also available we used the latter because this is the variation that a choosing potential mate is confronted with.

### Meta-analytic Technique

Effect sizes were expressed as Pearson’s *r* and were either directly extracted, calculated from statistics reported using the appropriate conversion formula [45] or measured from graphs (using ImageJ [55]). Pearson’s *r*’s were transformed to Fisher’s *Zr*’s before analysis [46]. These effect sizes were weighted using the total sample size (N) –3 [46]. When effect sizes were calculated from statistics where only the degrees of freedom (DF) were reported we used N = DF+2. To correct for statistical non-independence of brood-mates, the number of broods rather than the number of nestlings measured was used as N.

Meta-analyses were performed using Bayesian mixed models implemented in MCMCglmm [56], [57] in R [58]. This approach is highly flexible and allows for the inclusion of study, species and phylogeny as random effects [47], [56]. Note that phylogeny was only included when the analysis contained more than three species (in practice only one analysis was run without phylogeny, H:L ratio against carotenoid level, table 1). Inverse Wishart priors were used (V = 1, nu = 0.002) and models were run three times each, with 10,000,000 iterations, burnin interval of 2,500,000 and thinning interval of 250. Convergence of the models was assessed using Gelman-Rubin statistics, which all were lower (except for the H:L ratio against carotenoid level model which probably included too few data per level to converge readily) than 1.05, which is lower than the recommended criterion of potential scale reduction of 1.1 among chains [59], [60].

**Table 1:** Overview of the parameters included in the meta-analyses, marked with x, per association investigated.

		Parameters									
Analysis		Phylogeny	Study	Species	Sex	Juvenile	Supplementation	Exp.variation	Assay	Carotenoidcolor	Site ofcoloration
traitredness	antibodyresponse	x	x	x				x			x
	H:L ratio	x	x	x							
	parasiteload	x	x	x	x						x
	PHAresponse	x	x	x		x		x			x
	whiteblood cells	x	x	x							
	antioxidantcapacity	x	x	x	x			x	x		x
	oxidativedamage	x	x	x				x	x		
carotenoidlevel	traitredness	x	x	x	x	x	x	x			x
	antibodyresponse	x	x	x	x		x				
	H:L ratio		x	x							
	parasiteload	x	x	x							
	PHAresponse	x	x	x	x	x	x	x		x	
	whiteblood cells	x	x	x		x					
	antioxidantcapacity	x	x	x	x	x	x	x	x	x	
	oxidativedamage	x	x	x	x	x	x	x	x	x	

See also data S1 and text for inclusion criteria of the moderators.

In several cases multiple effect sizes were extracted from one study and/or from the same species, and controlling for the non-independence of these effect sizes yields a more precise estimate of the effects and their confidence limits. This approach can be considered conservative when compared to treating each effect size estimate as an independent data point. The phylogeny included was a pruned supertree of birds [61]. This is a maximum parsimony tree and therefore without estimates of branch lengths. To date no comparable supertree of birds is available with estimates of branch lengths, therefore we assumed equal branch lengths and scaled these to obtain an ultrametric tree. Branch lengths between nodes thus equaled one divided by the number of nodes from root to tip.

Publication bias can be a potential caveat in meta-analysis given that the tendency not to publish non-significant relationships can inflate average effect sizes [62]. These biases become apparent in asymmetry of a funnel plot, in which effects sizes are plotted against the corresponding sample sizes (funnel plots S1). Additionally, publication bias is less likely to be present when data is obtained from the authors directly when not all statistics of interest to a meta-analysis were reported. This was the case in 25% of the effect sizes included in the present study (data S1). The rank correlation test for funnel plot asymmetry [48] did not reach significance in any of the analyses (all p>0.05).

### Moderators

Effect sizes can differ between studies for many reasons, including stochastic variation, but also potential moderating variables. Therefore, we included several moderating variables to test whether they explain variation in effect sizes. In each separate meta-analysis we only included moderators for which we had at least three effect size estimates per level (data S1, table 1). These moderators were added simultaneously to the corresponding models. For moderators that showed at least a trend (p<0.1), separate meta-analyses were run within the levels of moderators to investigate overall effects sizes in these subcategories. For example, when sex showed a trend in the overall model, we ran separate models for each sex. We chose to investigate trends in addition to significant effects for two reasons: i) to be conservative in controlling for potential confounding variables and ii) because multi-level moderators with a low number of effect sizes per level may be hard to detect, but may provide new testable hypotheses.

We included the following moderators: i) Sex, for which we coded unknown sex as 0, females as −1, and males as 1 ii) Whether adults or juveniles were studied. iii) Whether carotenoids were supplemented or not. iv) Whether the effect size was subject to experimental variation, caused by treatments other than carotenoid supplementation, which potentially increased variation in the traits of interest. To avoid such effects we selected pre-experimental (including carotenoid supplementation studies) values or results of analyses of the control group only, when possible. v) The oxidative stress state assay used. For antioxidant capacity the following levels were distinguished: the OXY test [63], the TEAC test [64] and the KRL test [65], which differ in components of the plasma antioxidant barrier that are measured [63]. For oxidative damage analyses the following moderator levels were dinstinguished: MDA, TBARS, [66] and d-ROM [67]. vi) In the analyses of the associations with carotenoid levels we included a moderator indicating whether the study species exhibited carotenoid-dependent coloration or not. Because the precise usage of carotenoids in pigmentation is unknown for many species, this was judged by the presence of yellow, orange or red traits characteristic for carotenoid-based pigmentation [51], [68]. Images were searched via Google Images with a search query that included genus and species name. The first nine pictures of the search results were viewed to be positive of the species identification and to control for variability in picture quality and coloration. Species for which these colors are known to be based on other pigments were coded as exhibiting no carotenoid pigmentation (e.g. *Gallus gallus* [69], [70]) or in a special case, the blue-footed booby, blue coloration was considered carotenoid based as this was previously demonstrated [71]. When carotenoids are used to pigment sexual traits one may expect higher effect sizes, given that sexual selection may increase variance in carotenoids due to investment into sexual traits. Additionally carotenoid-based signals are perhaps more likely to evolve in species in which carotenoids play a major physiological role. vii) Within the sexual signal analyses, we included a moderator indicating whether the carotenoid-dependent coloration was expressed in plumage or other tissue (e.g. the bill), using the same pictures as described above. Plumage pigmentation reflects physiological state at molt and may therefore signal current physiological state less reliably than carotenoid-dependent coloration in other tissues, such as the bill, that can change more rapidly.

### Color

Aspects of light reflectance together composing the perception of variation in colors are described in many ways, for example brightness, hue and chroma [72] or principal components [73]. These aspects can also be captured in various ways, for example by comparing color charts [74], digital photography [75], [76] or spectrophotometry [73]. Not surprisingly, studies from which we extracted effect sizes related to coloration used different descriptions of color. Effect sizes of these studies were interpreted as follows: i) Measures which corresponded to a shifted weighted spectrum towards red were interpreted as representing increasing carotenoid content in a trait. This includes a shift in the shape of the curve towards the red part of the spectrum (corresponding to hue) and an increase in the relative reflectance in the red part of the spectrum (corresponding to chroma). Papers with color measures that corresponded to total reflectance (brightness) could not be interpreted as either increasing or decreasing carotenoid content and were not included, except when the authors presented evidence of it reflecting carotenoid content of the trait considered. ii) When multiple relationships of the considered (see above) color metrics were reported we took an average of effect sizes across these metrics. The sign of the effect size was expressed as positive when the relationship showed a positive relationship with carotenoid-dependent color intensity (i.e. trait redness).

### Immune System Components

The immune system is complex, and several components of the immune system have been studied in relation to carotenoid levels and carotenoid-dependent coloration. In our analyses we considered the measures of the immune system of which we found four or more independent studies. These measures were as follows: PHA response, antibody production against experimentally induced antigens, parasite load and white blood cell counts.

Swelling induced by the subcutaneous injection of phytohaemagglutinin (PHA, a lectin found in plants used as mitogen) is a widely used test in birds and other vertebrates [77]. Larger swellings are interpreted as a stronger immune response, a view supported by the finding that larger swellings are usually found in individuals or experimental groups that can be considered to be in a better state [77]–[80]. However, the specific immunology behind PHA responses in birds is still debatable possibly limiting such straightforward interpretation [81]. Especially the common interpretation that PHA responses represent a T-cell mediated immune response alone may be incomplete [78], [79].

Antibody responses are commonly assumed to be more effective with increasing amount of antibodies produced [82]–[84]. We did not discriminate between the different antigens used to induce an immune response, because several antigens were only used in one or a few studies (see data S1).

White blood cell counts are more difficult to interpret, because they can both indicate current infections or high immunocompetence. Separate populations of white blood cells may be abundant because of a current infection or higher levels may indicate the ability to launch a more potent immune response [82]. We analyzed studies reporting on separate types of white blood cells together and averaged correlations reported with our variables of interest across separate types of white blood cells when they were reported within a single study to make the most of the available data. Higher ratios of heterophils over lymphocytes are considered a reliable indicator of higher stress [85], which we therefore analyzed in a separate analysis.

Parasite infection may either reflect inability to clear parasites or the ability to tolerate parasites [86]. In the case of carotenoid-dependent coloration, resource allocation of carotenoids towards signal intensity is either predicted to increase parasite load in bright males, or, when resource availability differs between individuals, parasite load is predicted to co-vary negatively with signal intensity [87]. A positive relationship between parasite load and signal intensity can also become apparent by selective disappearance of highly parasitized low-quality individuals harboring signals of low intensity [88].

### Oxidative Stress State

The imbalance between the production of free radicals and antioxidant defenses that quench them is termed oxidative stress [29]. Free radical damage to “crucial” cell components can impair physiological function and it is this impairment that is viewed as a major agent of senescence [29]. To capture aspects of oxidative stress, measures of antioxidant defense and oxidative damage are employed [30]. To interpret differences in oxidative stress state between individuals both these measures are required [30]. When for example antioxidant capacity increases in response to increased exposure to free radicals, conclusions based solely on either antioxidant capacity or oxidative damage will lead to opposite conclusions (e.g. antioxidant capacity is higher, indicating lower oxidative stress; oxidative damage is higher indicating higher oxidative stress). Therefore we performed separate meta-analyses for effect sizes of antioxidant capacity and oxidative damage.

Assuming that carotenoids do not directly increase free radical production an association of carotenoid level with higher antioxidant capacity is likely to reflect a positive effect of carotenoids on resistance against free radicals. This is expected to also result in lowered oxidative damage measured, possibly depending on the composition of antioxidant defenses and the proxy of oxidative damage measured. We therefore speculate that associations of carotenoids with decreased measured oxidative damage or increased antioxidant capacity can be interpreted as a reduction in oxidative stress, improving oxidative stress state. Associations with trait redness are more elusive, because it cannot be excluded that free radical production is associated with trait expression.

### Carotenoids

There are many subtypes of carotenoids and species differ in the combinations of carotenoids they incorporate into sexual traits (reviewed in [49]) as well as in the relative levels in plasma [89]. Because of the diversity of ways that carotenoid levels are reported discrimination between different types of carotenoids was not feasible in our meta-analyses, and we pooled separate correlations between levels of carotenoid subtypes and the variables of interest when they were reported separately. In many studies carotenoid levels in plasma are assessed colorimetrically which only yields a total plasma concentration of carotenoids (e.g. [33]). Additionally, information on the precise mechanisms of specific carotenoid incorporation in pigmentation is lacking for many species [49] and carotenoids are likely metabolized into different subtypes [49], [90], [91]. Hence a more detailed treatment of carotenoid levels was not feasible, even though we recognize that this could provide more informative estimates.

### Differences between Species

Relationships between different antioxidants, including carotenoids, and antioxidant capacity vary substantially across species [89]. Sexual selection for carotenoid-dependent coloration may also differ between species. In some species carotenoid-dependent coloration may not have evolved into a costly signal that advertises condition, may be a remnant of past selection, or may serve other roles such as sex and species recognition [1], [92]. To assess the importance of interspecific variation in our meta-analyses we first compared whether adding species and phylogeny to a model which included only study improved the model, as judged by the deviance information criterion (DIC) (table 2). Lower DIC values indicate a better fit and can be considered the Bayesian counterpart of the Akaike information criterion (AIC) [60]. Additionally we calculated the proportion of heterogeneity explained by species and phylogeny in the model [47], [60]. To visualize some of these differences between species and directly test within-species effects we performed within-species meta-analyses within the datasets for which we found at least three separate studies per single species. These analyses were performed using the metafor package [48] in R [58] using random-effects meta-analysis estimated using REML, given that the complex data structure for which we employed MCMCglmm is not present within species. Multiple effect sizes per study were pooled by using a weighted average for sample size.

**Table 2:** Overview of the separate meta-analyses performed.

			Results (* marks significance)			DIC (* marks improved model)		Heterogeneity (%)	
Analysis			*r* (95% CI)	ESs	species	study only	full model	species and phylogeny	residual
trait redness	immune system	antibody response	−0.03 (−0.37,0.32)	10	6	−15.77	−14.19	5	47
		H:L ratio	0.00 (−0.52,0.51)	9	6	1.28	−5.02*	2	7
		parasite load	−0.01 (−0.19,0.19)	23	15	−11.39	−32.26*	74	14
		PHA response	0.17 (0.02,0.31)*	22	15	−37.29	−37.69*	8	28
		white blood cell count	−0.05 (−0.49,0.42)	8	6	−1.72	−7.03*	4	5
	oxidative stress	antioxidant capacity	−0.01 (−0.19,0.17)	19	9	−43.35	−42.17	10	17
		oxidative damage	−0.11 (−0.35,0.12)	14	8	−25.25	−28.92*	12	7
carotenoid level	color	trait redness (all)	0.35 (0.28,0.42)*	83	28	15.10	16.59	4	64
		trait redness (males)	0.37 (0.29,0.46)*	45	21	−53.46	−51.96	6	61
		trait redness (females)	0.26 (0.08,0.43)*	17	15	−19.40	−26.35*	7	9
		trait redness (all without supplementation)	0.28 (0.20,0.37)*	55	19	0.45	−1.34*	8	50
	immune system	antibody response	0.11 (−0.15,0.35)	14	7	−27.59	−28.28*	10	15
		H:L ratio	−0.04 (−0.46,0.44)	5	3	−12.08	−12.13*	1	10
		parasite load	−0.23 (−0.45,0.02)	8	6	−20.53	−20.13	11	10
		PHA response	0.20 (0.01,0.38)*	34	19	−31.62	−35.97*	8	57
		white blood cell count	−0.10 (−0.35,0.19)	11	8	−22.10	−25.17*	20	5
	oxidative stress	antioxidant capacity	0.10 (0.01,0.19)*	64	37	−156.13	−176.54*	13	7
		oxidative damage	−0.02 (−0.14,0.09)	33	15	−79.92	−77.5	7	13
		oxidative damage (males)	−0.1 (−0.25,0.06)	17	10	−40.41	−39.37	10	10

### Residual Heterogeneity

Heterogeneity between effect sizes due to factors other than the moderators described above can suggest potential for additional moderators to explain variation among effect sizes. We calculated the residual heterogeneity according to Nakagawa and Santos [47]. The variance component of study and the residual variance were summed per sample along the chain and divided by the sum of all variance components and the typical sampling error variance [47] to calculate the proportion of residual heterogeneity. Low, moderate and high levels of heterogeneity are considered to be 25%, 50% and 75% respectively (equations 22–25 in [47]).

## Results

Our literature search identified 148 studies [22], [23], [25], [35], [54], [64], [67], [71], [89], [93]–[229] on 88 species with information on 357 estimates of effect sizes falling into 15 categories of pairwise associations among relevant variables (data S1).

### Carotenoids, Trait Redness

**Figure 2:**
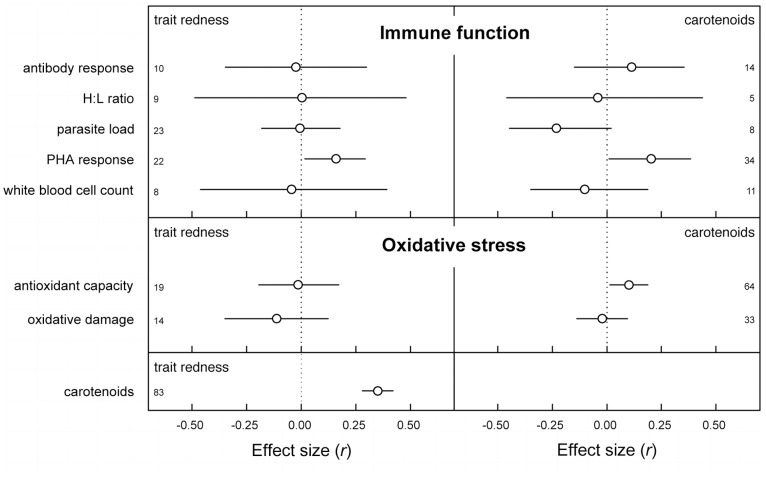
Effect sizes (±95% confidence interval) of the main 15 meta-analyses. Note that when the confidence interval does not intersect the dotted line (which reflects *r* = 0), the effect size is statistically significant. Numbers along the y-axis depict the number of effect sizes included in each analysis. The variables along the y-axis depict the relationships analyzed with the variables depicted in the upper corners of each panel.

As expected we found that carotenoid availability was positively related to redness of sexual traits (figure 2, p<0.0001). Experiments that supplemented carotenoids rather than correlations with concentrations of carotenoids in blood resulted in significantly larger effect sizes (p = 0.006), but an analysis without the supplementation studies still yielded a highly significant overall effect size (table 2, p<0.0001). Males tended to show higher effect sizes than females (sex, p = 0.094), though both sexes showed significant associations in stratified analyses (table 2, p<0.01).

### Immune Function

Of the five categories of immunological measurements only the PHA response was significantly and positively associated with higher carotenoid levels (p = 0.04) and trait redness (p = 0.03). We detected a tendency for higher carotenoid levels to be associated with lower parasite load (figure 2, p = 0.06). This was however not reflected in the relationship between trait redness and parasite load (p = 0.93). We did not detect any effects of the moderating variables included (table 1).

### Oxidative Stress State

Carotenoid levels increased with antioxidant capacity (figure 2, p = 0.027), but were not associated with oxidative damage (table 2, p = 0.70). The relationships of trait redness with antioxidant capacity (p = 0.86) and oxidative damage (p = 0.30) were not significant (figure 2). Only for the relationship between carotenoid levels and oxidative damage did we detect any effect of the moderators included (table 1, table 2). Males tended to have a lower effect size (p = 0.097), and within males we did not detect a significant overall effect size (p = 0.20).

### Differences between Species

**Figure 3:**
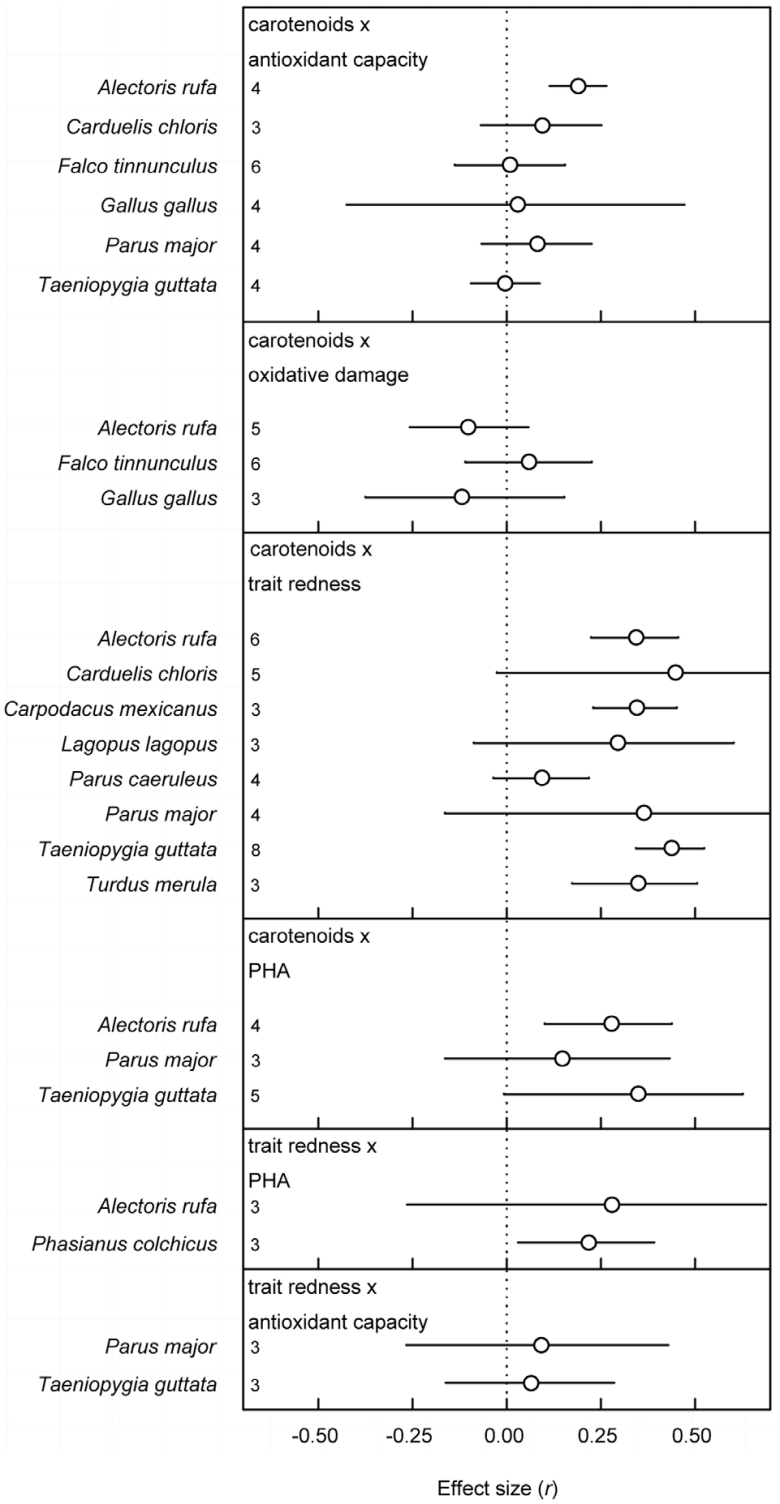
Plot of separate overall effect sizes (±95% confidence interval) per species. An overall effect size per species was only calculated when three or more studies were available per relationship of interest. Numbers along the y-axis depict the number of effect sizes included in each overall effect size.

Of the 17 models, the within-moderator level analyses, 12 models improved when species and phylogeny were added to the model (table 2, as judged by reduction in DIC). This demonstrates variation between species over and above variation attributable to differences between studies or typical sampling error variance. Separate analyses of species for which three or more effect sizes were available also showed considerable variation (figure 3), but note that the analyses presented in table 2 are with all species included. The separate analyses (figure 3) also show that in some species significant overall effects can be detected which are in accordance with the overall effects (figure 2), whereas in other species they are not, further illustrating the heterogeneity between species.

### Causality

If carotenoid levels covary with the physiological variables we summarized here without being causally involved, inclusion of studies that supplement carotenoids would lower effect sizes. For the relationship of carotenoid levels with both antioxidant capacity and PHA response we found positive non-significant associations with the supplementation moderator (estimate = 0.14 (−0.06∶0.34 95% CI), p = 0.15 and estimate = 0.13 (−0.23∶0.51, 95% CI), p = 0.48 respectively). That these associations are non-significant implies that the effects of natural and experimental variation cannot be distinguished, indicating that carotenoids are likely to be mechanistically involved. Carotenoid supplementation was associated with higher effect sizes of the increase in trait redness (see above). This suggests that either the dosages used induce carotenoid levels outside of the normal range or that it decreases variance between individuals, both can increase effect size.

## Discussion

**Figure 4:**
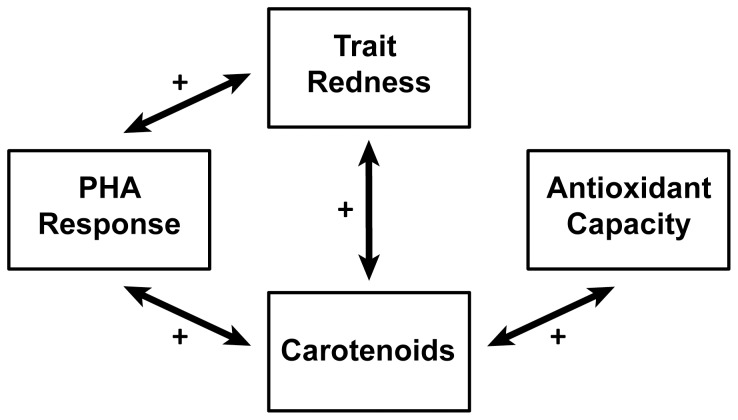
Overview of the significant overall effect sizes framed in the layout of figure 1. A full circle argument can only be made for the relationship of carotenoids and trait redness with PHA response. Carotenoids do however signal lowered antioxidant capcity, but there was no significant relationship with trait redness. Plusses and minuses indicate positive or negative relationships.

Pooling a large number of studies through meta-analysis, we found evidence for the hypothesized honesty maintenance mechanisms of the associations of carotenoid levels with immune functioning and oxidative stress state (figures 2 and 4). Honest signaling via carotenoids was only apparent in PHA response, given that carotenoids and trait redness were both associated with a greater swelling in response to PHA injection. Carotenoids tended to be associated with lower parasite abundance suggesting that carotenoids may signal multiple components of the immune system; however, this effect was not mimicked in the association with trait redness. Future studies may reveal whether carotenoids directly increase the efficacy of components of the immune system involved in the PHA response. Alternatively, carotenoids may alter oxidative stress state which may in turn affect immune responses [37], [40]–[42].

For oxidative stress state, we show that carotenoids signal higher antioxidant capacity, increased resistance against free radicals. However no association between trait redness and oxidative stress state was found (figure 2), and hence we find no evidence that antioxidant capacity is signaled via carotenoid-dependent traits. The size of the overall effect is modest suggesting that carotenoids are indeed only minor contributors to antioxidant capacity *in vivo* as suggested previously [27], [28], [89], [230]. However the significance of the overall effect size does show that carotenoids can signal oxidative stress state in birds, and our finding that the effect size was independent of whether carotenoids were experimentally supplemented or not suggests this relationship to be causal. Moreover, carotenoids may be more important in regulating oxidative balance in other parts of the body than in the blood circulation, such as cell membranes, information not necessarily captured by the plasma carotenoid levels we used in this study.

An alternative explanation for the modest overall effect sizes we find is that carotenoid levels are required for retinoid production or indicate the amount of free radicals that are not quenched by other antioxidant machinery (i.e. enzymatic and non-enzymatic) [27]. In this scenario carotenoids are not contributing substantially to the quenching of free radicals but are damaged (i.e. bleached) by them. A correlation between carotenoids and oxidative stress state is then still expected, but may be weak. Under this scenario, the total carotenoid store in the body could be viewed as dynamic indicator of past levels of free radicals that were not quenched by other antioxidants. Measures of antioxidant capacity and oxidative damage are relatively flexible within an individual as demonstrated by their modest within individual repeatability. These repeatabilities in birds range from 0.12 (Beamonte-Barrientos & Verhulst submitted), 0.14 [231], 0.3 [127] to 0.49 [232] for antioxidant capacity and 0.18 [231], 0.42 (Beamonte-Barrientos & Verhulst submitted) to 0.60 [127] for oxidative damage. Correlations with these flexible parameters are therefore predicted to be weak when the carotenoid store integrates past damage and is therefore both lagging and less flexible. The same reasoning holds for the redness of carotenoid-dependent sexual traits, which are considered relatively consistent and have also been experimentally shown to lag in response to immune challenges [53], [106]. In addition, variation between studies in the assays used for antioxidant capacity [63] and oxidative damage assays [233] used may weaken the relationship between carotenoids and oxidative stress state. However we did not detect a moderating effect of assay method.

The interspecific variation that was apparent in most analyses (table 2) can also result in lower overall effect sizes. When in some species carotenoid levels and trait redness signal different aspects of physiology or are physiologically less important, effect sizes across species are expected to be lower. Also in some species carotenoid-dependent coloration may not currently be under sexual selection or choosiness for these traits may differ between species. Ideally, we would want to relate the preference for carotenoid-dependent coloration per species to the differences in effect sizes we report. However for carotenoid-dependent traits relatively few species (reviewed in [234], 15 species with little overlap with the species in this study) have been studied in mate-choice experiments and there is only one quantitative meta-analysis within a species, the zebra finch [16]. Given that mate-choice experiments are difficult to conduct or variable in general, judging from its repeatability [235], this may prove difficult.

Interestingly, there is no single species in which all effect sizes that were overall significant were also significant within that species (figure 3). The study of carotenoid-dependent signaling may thus profit from both in-depth study of a single species, and by examining different species or explaining between-species variation. Which carotenoids are used in pigmenting their traits or how they metabolize or sequester carotenoids may be key. Residual heterogeneity was low in general (table 2) except for the correlations of carotenoid levels with PHA response and trait redness in which residual heterogeneity was moderate. The latter may be due to the differences between species in carotenoid usage. This may also be a reason why some of the between species moderators we examined in this study failed to reach significance.

Whether or not a species evolved carotenoid-dependent coloration as a signal may depend, among other things, on the importance of carotenoids in its physiology, or on the scarcity of carotenoids in the environment, and such effects could be reflected in the relationships between carotenoids on the one hand, and aspects of immune function and oxidative stress state on the other hand. However, whether or not a species exhibited carotenoid-dependent coloration did not affect effect size estimates in our analyses. This may suggest that carotenoids serve the same physiological functions in species exhibiting carotenoid-dependent coloration and those lacking them. Additionally it suggests that the increase in variance caused by sexual selection for carotenoid incorporation into sexual coloration is too small or variable to detect. This may also be the reason why we only detected two trends of lowered effect sizes in females, the generally choosier sex. Within the analyses on trait redness we also did not detect a moderating effect of whether the trait was plumage related and thus subject to molting patterns. A reduction in effect size was expected given that plumage can only signal carotenoid content at molt and is therefore less flexible than for instance skin coloration [236]. The lack of such an association might actually be a reflection of how long past oxidative stress is encoded into carotenoid stores and sexual traits, but this awaits future study.

The two aspects of physiology associated with carotenoids we summarized here, immune function and oxidative stress state, may help maintain honesty of carotenoid-based signals. The use of carotenoids in sexual traits diverts carotenoids away from its benefit for immune function and antioxidant capacity creating a handicap. These aspects are not mutually exclusive with the hypothesis that carotenoid levels integrate information on past exposure to free radicals not quenched by other more potent antioxidants or antioxidant machinery. All three mechanisms have in common that a larger supply of carotenoids into the body will yield a more colorful sexual ornament, increasing mating success. The sequestering of carotenoids, in terms of foraging, assimilation, transport abilities or the like, is under positive selective pressure. This may still be the major mechanism maintaining honesty, together with the beneficial physiological functions of carotenoids, which may be indicative of shorter-term history. Examining how large the contributions of carotenoid sequestering on the one hand and carotenoid use within the body on the other hand are in determining honesty of carotenoid-dependent coloration will require the direct measurement of sequestration and carotenoid turnover, preferably also under both immunological and oxidative stress. This will be exciting yet experimentally challenging. It seems the more complete our understanding of carotenoid-dependent signaling becomes the more areas of biology become involved. The integrated involvement of carotenoids in major physiological areas in combination with their light absorbing properties may be why carotenoid-based sexual signals are common.

## Supporting Information

Funnel Plots S1 Plotted in the order of table 1 and data S1 are the separate datasets of effect sizes plotted against their corresponding sample sizes. The title depicts the relationship plotted.
(PDF)

Data S1 Excel file of the datasets collected for the separate meta-analyses.
(XLSX)

PRISMA S1 PRISMA flow diagram showing the literature search procedure.
(PDF)

PRISMA S2 PRISMA checklist for meta-analyses.
(PDF)

## References

[1] Andersson M, Iwasa Y (1996) Sexual selection. Trends Ecol Evol 11: 53–58. 21237761 10.1016/0169-5347(96)81042-1

[2] Fisher RA (1930) The genetical theory of natural selection. Oxford, UK: Clarendon Press. 272 p.

[3] Maan ME, Seehausen O (2011) Ecology, sexual selection and speciation. Ecology Letters 14: 591–602. 21375683 10.1111/j.1461-0248.2011.01606.x

[4] Zahavi A (1975) Mate selection: a selection for a handicap. J Theor Biol 53: 205–214. 1195756 10.1016/0022-5193(75)90111-3

[5] Grafen A (1990) Biological signals as handicaps. J Theor Biol 144: 517–546. 2402153 10.1016/s0022-5193(05)80088-8

[6] Kotiaho JS (2001) Costs of sexual traits: a mismatch between theoretical considerations and empirical evidence. Biol Rev 76: 365–376. 11569789 10.1017/s1464793101005711

[7] Kokko H, Jennions MD, Brooks R (2006) Unifying and testing models of sexual selection. Annual Reviews 37: 43–66.

[8] Számadó S (2011) The cost of honesty and the fallacy of the handicap principle. Anim Behav 81: 3–10.

[9] Hill GE (2011) Condition-dependent traits as signals of the functionality of vital cellular processes. Ecology Letters 14: 625–634. 21518211 10.1111/j.1461-0248.2011.01622.x

[10] Schantz von T, Bensch S, Grahn M, Hasselquist D, Wittzell H (1999) Good genes, oxidative stress and condition–dependent sexual signals. Proc R Soc B 266: 1–12. 10.1098/rspb.1999.0597 PMC1689644 10081154

[11] Lozano GA (1994) Carotenoids, parasites, and sexual selection. Oikos 70: 309–311.

[12] Olson V, Owens I (1998) Costly sexual signals: are carotenoids rare, risky or required? Trends Ecol Evol 13: 510–514. 21238418 10.1016/s0169-5347(98)01484-0

[13] Kemp DJ, Herberstein ME, Grether GF (2012) Unraveling the true complexity of costly color signaling. Behav Ecol 23: 233–236.

[14] Svensson PA, Wong BBM (2011) Carotenoid-based signals in behavioural ecology: a review. Behaviour 148: 131–189.

[15] Vinkler M, Albrecht T (2010) Carotenoid maintenance handicap and the physiology of carotenoid-based signalisation of health. Naturwissenschaften 97: 19–28. 19680618 10.1007/s00114-009-0595-9

[16] Simons MJP, Verhulst S (2011) Zebra finch females prefer males with redder bills independent of song rate–a meta-analysis. Behav Ecol 22: 755–762.

[17] Sundberg J (1995) Female Yellowhammers (Emberiza-Citrinella) Prefer Yellower Males - a Laboratory Experiment. Behav Ecol Sociobiol 37: 275–282.

[18] Jawor JM, Linville S, Beall S, Breitwisch R (2003) Assortative mating by multiple ornaments in northern cardinals (Cardinalis cardinalis). Behav Ecol 14: 515–520.

[19] Toomey MB, McGraw KJ (2012) Mate choice for a male carotenoid-based ornament is linked to female dietary carotenoid intake and accumulation. BMC Evol Biol 12: 3. 22233462 10.1186/1471-2148-12-3 PMC3315416

[20] Künzler R, Bakker T (2001) Female preferences for single and combined traits in computer animated stickleback males. Behav Ecol 12: 681–685.

[21] Kodric-Brown A (1989) Dietary carotenoids and male mating success in the guppy: an environmental component to female choice. Behav Ecol Sociobiol 25: 393–401.

[22] Blount JD, Metcalfe NB, Birkhead TR, Surai PF (2003) Carotenoid modulation of immune function and sexual attractiveness in zebra finches. Science 300: 125–127. 12677066 10.1126/science.1082142

[23] McGraw KJ, Ardia DR (2003) Carotenoids, immunocompetence, and the information content of sexual colors: an experimental test. Am Nat 162: 704–712. 14737708 10.1086/378904

[24] Pike TW, Blount JD, Bjerkeng B, Lindström J, Metcalfe NB (2007) Carotenoids, oxidative stress and female mating preference for longer lived males. Proc R Soc B 274: 1591–1596. 10.1098/rspb.2007.0317 PMC2169282 17439854

[25] Karu U, Saks L, Hõrak P (2008) Carotenoid-based plumage coloration is not affected by vitamin E supplementation in male greenfinches. Ecol Res 23: 931–935.

[26] Yeuhm K, Aldini G, Russell RM, Krinsky NI (2009) Antioxidant/Pro-oxidant actions of carotenoids. In: Britton G, Liaaen-Jensen S, Pfander H, editors. Carotenoids: volume 5: nutrition and health. 235–268.

[27] Hartley RC, Kennedy MW (2004) Are carotenoids a red herring in sexual display? Trends Ecol Evol 19: 353–354. 16701285 10.1016/j.tree.2004.04.002

[28] Costantini D, Møller AP (2008) Carotenoids are minor antioxidants for birds. Funct Ecol 22: 367–370.

[29] Finkel T, Holbrook NJ (2000) Oxidants, oxidative stress and the biology of ageing. Nature 408: 239–247. 11089981 10.1038/35041687

[30] Costantini D, Verhulst S (2009) Does high antioxidant capacity indicate low oxidative stress? Funct Ecol 23: 506–509.

[31] Monaghan P, Metcalfe NB, Torres R (2009) Oxidative stress as a mediator of life history trade-offs: mechanisms, measurements and interpretation. Ecology Letters 12: 75–92. 19016828 10.1111/j.1461-0248.2008.01258.x

[32] Peters A (2007) Testosterone and carotenoids: an integrated view of trade-offs between immunity and sexual signalling. BioEssays 29: 427–430. 17450573 10.1002/bies.20563

[33] Bertrand S, Faivre B, Sorci G (2006) Do carotenoid-based sexual traits signal the availability of non-pigmentary antioxidants? J Exp Biol 209: 4414–4419. 17079711 10.1242/jeb.02540

[34] Pike TW, Blount JD, Lindström J, Metcalfe NB (2007) Availability of non-carotenoid antioxidants affects the expression of a carotenoid-based sexual ornament. Biol Lett 3: 353–356. 17472903 10.1098/rsbl.2007.0072 PMC2390655

[35] Pérez C, Lores M, Velando A (2008) Availability of nonpigmentary antioxidant affects red coloration in gulls. Behav Ecol 19: 967–973.

[36] Pérez-Rodríguez L (2009) Carotenoids in evolutionary ecology: re-evaluating the antioxidant role. BioEssays 31: 1116–1126. 19705366 10.1002/bies.200900070

[37] Costantini D, Møller AP (2009) Does immune response cause oxidative stress in birds? A meta-analysis. Comp Biochem Phys A 153: 339–344. 10.1016/j.cbpa.2009.03.010 19303455

[38] de la Fuente M (2002) Effects of antioxidants on immune system ageing. Eur J Clin Nutr 56: S5–S8. 12142953 10.1038/sj.ejcn.1601476

[39] de la Fuente M, Victor VM (2000) Anti-oxidants as modulators of immune function. Immunol Cell Biol 78: 49–54. 10651929 10.1046/j.1440-1711.2000.00884.x

[40] Bendich A (1989) Carotenoids and the immune response. J Nutr 119: 112–115. 2643693 10.1093/jn/119.1.112

[41] Hughes DA (1999) Effects of carotenoids on human immune function. P Nutr Soc 58: 713–718. 10.1017/s0029665199000932 10604207

[42] Chew BP, Park JS (2004) Carotenoid action on the immune response. J Nutr 134: 257S–261S. 14704330 10.1093/jn/134.1.257S

[43] Garbe A (1992) Retinoids are important cofactors in T cell activation. J Exp Med 176: 109–117. 1535365 10.1084/jem.176.1.109 PMC2119299

[44] Semba RD (1998) The role of vitamin A and related retinoids in immune function. Nutr Rev 56: S38–S48. 10.1111/j.1753-4887.1998.tb01643.x 9481123

[45] Rosenthal R (1994) Parametric measures of effect size. In: Cooper H, Hedges L, editors. The handbook of research synthesis. New York: Russell Sage Foundation Publications. 231–244.

[46] Nakagawa S, Cuthill IC (2007) Effect size, confidence interval and statistical significance: a practical guide for biologists. Biol Rev Camb Philos Soc 82: 591–605. 17944619 10.1111/j.1469-185X.2007.00027.x

[47] Nakagawa S, Santos (2012) Methodological issues and advances in biological meta-analysis. Evolutionary Ecology: In press.

[48] Viechtbauer W (2010) Conducting Meta-Analyses in R with the metafor Package. Journal of Statistical Software 36: 1–48.

[49] McGraw KJ (2006) Mechanics of carotenoid-based coloration. In: Hill GE, McGraw KJ, editors. Bird coloration: Mechanisms and Measurements. Cambridge: Harvard University Press. 177–242.

[50] McGraw KJ (2004) Colorful songbirds metabolize carotenoids at the integument. J Avian Biol 35: 471–476.

[51] Olson VA, Owens IPF (2005) Interspecific variation in the use of carotenoid-based coloration in birds: diet, life history and phylogeny. J Evol Biol 18: 1534–1546. 16313466 10.1111/j.1420-9101.2005.00940.x

[52] Liberati A, Altman DG, Tetzlaff J, Mulrow C, Gøtzsche PC, et al (2009) The PRISMA statement for reporting systematic reviews and meta-analyses of studies that evaluate health care interventions: explanation and elaboration. J Clin Epidemiol 62: e1–e34. 19631507 10.1016/j.jclinepi.2009.06.006

[53] Faivre B, Grégoire A, Préault M, Cézilly F, Sorci G (2003) Immune activation rapidly mirrored in a secondary sexual trait. Science 300: 103–103. 12677062 10.1126/science.1081802

[54] Alonso-Álvarez C, Galván I (2011) Free radical exposure creates paler carotenoid-based ornaments: a possible interaction in the expression of black and red traits. PLoS ONE 6: e19403. 21556328 10.1371/journal.pone.0019403 PMC3083443

[55] Abràmoff MD, Magalhães PJ, Ram SJ (2004) Image processing with ImageJ. Biophotonics international 11: 36–42.

[56] Hadfield JD, Nakagawa S (2010) General quantitative genetic methods for comparative biology: phylogenies, taxonomies and multi-trait models for continuous and categorical characters. J Evol Biol 23: 494–508. 20070460 10.1111/j.1420-9101.2009.01915.x

[57] Hadfield JD (2010) MCMC Methods for Multi-Response Generalized Linear Mixed Models: The MCMCglmm R Package. Journal of Statistical Software 33: 1–22. 20808728 PMC2929880

[58] R Development Core Team (2011) R: A Language and Environment for Statistical Computing. R Foundation for Statistical Computing, Vienna, Austria.

[59] Gelman A, Rubin DB (1992) Inference from iterative simulation using multiple sequences. Statistical science 7: 457–472.

[60] Horváthová T, Nakagawa S, Uller T (2012) Strategic female reproductive investment in response to male attractiveness in birds. Proc R Soc B: In press. 10.1098/rspb.2011.0663 PMC3223647 21632630

[61] Davis KE (2008) Reweaving the tapestry: a supertree of birds. PhD thesis, University of Glasgow. 10.1371/currents.tol.c1af68dda7c999ed9f1e4b2d2df7a08e PMC4055607 24944845

[62] Begg CB, Berlin JA (1988) Publication Bias - a Problem in Interpreting Medical Data. J Roy Stat Soc a Sta 151: 419–463.

[63] Costantini D (2011) On the measurement of circulating antioxidant capacity and the nightmare of uric acid. Methods in Ecology and Evolution 2: 321–325.

[64] Cohen AA, Klasing K, Ricklefs R (2007) Measuring circulating antioxidants in wild birds. Comp Biochem Phys B 147: 110–121. 10.1016/j.cbpb.2006.12.015 17303461

[65] Alonso-Álvarez C, Bertrand S, Devevey G, Prost J, Faivre B, et al (2004) Increased susceptibility to oxidative stress as a proximate cost of reproduction. Ecology Letters 7: 363–368.

[66] Monaghan P, Metcalfe NB, Torres R (2009) Oxidative stress as a mediator of life history trade-offs: mechanisms, measurements and interpretation. Ecology Letters 12: 75–92. 19016828 10.1111/j.1461-0248.2008.01258.x

[67] Costantini D, Casagrande S, De Filippis S, Brambilla G, Fanfani A, et al (2006) Correlates of oxidative stress in wild kestrel nestlings (Falco tinnunculus). J Comp Physiol B 176: 329–337. 16344989 10.1007/s00360-005-0055-6

[68] Gray DA (1996) Carotenoids and sexual dichromatism in North American passerine birds. Am Nat 148: 453–480.

[69] McGraw KJ, Wakamatsu K, Ito S, Nolan PM, Jouventin P, et al (2004) You can’t judge a pigment by its color: carotenoid and melanin content of yellow and brown feathers in swallows, bluebirds, penguins, and domestic chickens. The Condor 106: 390–395.

[70] McGraw KJ, Klasing KC (2006) Carotenoids, immunity, and integumentary coloration in red junglefowl (Gallus gallus). The Auk 123: 1161–1171.

[71] Velando A, Beamonte-Barrientos R, Torres R (2006) Pigment-based skin colour in the blue-footed booby: an honest signal of current condition used by females to adjust reproductive investment. Oecologia 149: 535–542. 16821015 10.1007/s00442-006-0457-5

[72] Endler JA (1990) On the measurement and classification of colour in studies of animal colour patterns. Biol J Linn Soc 41: 315–352.

[73] Butler MW, Toomey MB, McGraw KJ (2011) How many color metrics do we need? Evaluating how different color-scoring procedures explain carotenoid pigment content in avian bare-part and plumage ornaments. Behav Ecol Sociobiol 65: 401–413.

[74] Burley N, Coopersmith CB (1987) Bill color preferences of zebra finches. Ethology 76: 133–151.

[75] Pike TW (2011) Using digital cameras to investigate animal colouration: estimating sensor sensitivity functions. Behav Ecol Sociobiol 65: 849–858.

[76] Stevens M, Parraga CA, Cuthill IC, Partridge JC, Troscianko TS (2007) Using digital photography to study animal coloration. Biol J Linn Soc 90: 211–237.

[77] Tella JL, Lemus JA, Carrete M, Blanco G (2008) The PHA Test Reflects Acquired T-Cell Mediated Immunocompetence in Birds. PLoS ONE 3: e3295. 18820730 10.1371/journal.pone.0003295 PMC2546448

[78] Martin LB, Han P, Lewittes J, Kuhlman JR, Klasing KC, et al (2006) Phytohemagglutinin-induced skin swelling in birds: histological support for a classic immunoecological technique. Funct Ecol 20: 290–299.

[79] Vinkler M, Bainová H, Albrecht T (2010) Functional analysis of the skin-swelling response to phytohaemagglutinin. Funct Ecol 24: 1081–1086.

[80] Verhulst S, Riedstra B, Wiersma P (2005) Brood size and immunity costs in zebra finches Taeniopygia guttata. J Avian Biol 36: 22–30.

[81] Kennedy MW, Nager RG (2006) The perils and prospects of using phytohaemagglutinin in evolutionary ecology. Trends Ecol Evol 21: 653–655. 17028055 10.1016/j.tree.2006.09.017

[82] Norris K, Evans MR (2000) Ecological immunology: life history trade-offs and immune defense in birds. Behav Ecol 11: 19–26.

[83] Deerenberg C, Arpanius V, Daan S, Bos N (1997) Reproductive effort decreases antibody responsiveness. Proc R Soc B 264: 1021–1029.

[84] Gross WG, Siegel PB, Hall RW, Domermuth CH, DuBoise RT (1980) Production and persistence of antibodies in chickens to sheep erythrocytes. 2. Resistance to infectious diseases. Poult Sci 59: 205–210. 6997852 10.3382/ps.0590205

[85] Davis AK, Maney DL, Maerz JC (2008) The use of leukocyte profiles to measure stress in vertebrates: a review for ecologists. Funct Ecol 22: 760–772.

[86] Råberg L, Sim D, Read AF (2007) Disentangling genetic variation for resistance and tolerance to infectious diseases in animals. Science 318: 812–814. 17975068 10.1126/science.1148526

[87] Shykoff JA, Widmer A (1996) Parasites and carotenoid-based signal intensity: how general should the relationship be? Naturwissenschaften 83: 113–121. 8622772 10.1007/BF01142175

[88] van de Pol M, Verhulst S (2006) Age-dependent traits: A new statistical model to separate within- and between-individual effects. Am Nat 167: 766–773. 16671020 10.1086/503331

[89] Cohen AA, McGraw KJ (2009) No simple measures for antioxidant status in birds: complexity in inter- and intraspecific correlations among circulating antioxidant types. Funct Ecol 23: 310–320.

[90] Stradi R, Pini E, Celentano G (2001) Carotenoids in bird plumage: the complement of red pigments in the plumage of wild and captive bullfinch (Pyrrhula pyrrhula). Comp Biochem Phys B 128: 529–535. 10.1016/s1096-4959(00)00353-5 11250548

[91] McGraw KJ, Hill GE, Stradi R, Parker RS (2001) The influence of carotenoid acquisition and utilization on the maintenance of species-typical plumage pigmentation in male American goldfinches (Carduelis tristis) and northern cardinals (Cardinalis cardinalis). Physiol Biochem Zool 74: 843–852. 11731975 10.1086/323797

[92] Candolin U (2003) The use of multiple cues in mate choice. Biol Rev 78: 575–595. 14700392 10.1017/s1464793103006158

[93] Aguilera E, Amat JA (2007) Carotenoids, immune response and the expression of sexual ornaments in male greenfinches (Carduelis chloris). Naturwissenschaften 94: 895–902. 17569027 10.1007/s00114-007-0268-5

[94] Alonso-Álvarez C, Bertrand S, Devevey G, Gaillard M, Prost J, et al (2004) An experimental test of the dose-dependent effect of carotenoids and immune activation on sexual signals and antioxidant activity. Am Nat 164: 651–659. 15540154 10.1086/424971

[95] Alonso-Álvarez C, Bertrand S, Devevey G, Prost J, Faivre B, et al (2006) An experimental manipulation of life-history trajectories and resistance to oxidative stress. Evolution 60: 1913–1924. 17089975

[96] Alonso-Álvarez C, Bertrand S, Faivre B, Chastel O, Sorci G (2007) Testosterone and oxidative stress: the oxidation handicap hypothesis. Proc R Soc B 274: 819–825. 10.1098/rspb.2006.3764 PMC2093982 17251089

[97] Alonso-Álvarez C, Pérez-Rodríguez L, García JT, Viñuela J (2009) Testosterone-mediated trade-offs in the old age: a new approach to the immunocompetence handicap and carotenoid-based sexual signalling. Proc R Soc B 276: 2093–2101. 10.1098/rspb.2008.1891 PMC2677252 19324780

[98] Alonso-Álvarez C, Pérez-Rodríguez L, Mateo R, Chastel O, Viñuela J (2008) The oxidation handicap hypothesis and the carotenoid allocation trade-off. J Evol Biol 21: 1789–1797. 18713241 10.1111/j.1420-9101.2008.01591.x

[99] Alonso-Álvarez C, Pérez-Rodríguez L, García JT, Viñuela J, Mateo R (2010) Age and breeding effort as sources of individual variability in oxidative stress markers in a bird species. Physiol Biochem Zool 83: 110–118. 19922287 10.1086/605395

[100] Arnold KE, Larcombe SD, Ducaroir L, Alexander L (2010) Antioxidant status, flight performance and sexual signalling in wild-type parrots. Behav Ecol Sociobiol 64: 1857–1866.

[101] Arriero EE, Fargallo JA (2006) Habitat structure is associated with the expression of carotenoid-based coloration in nestling blue tits Parus caeruleus. Naturwissenschaften 93: 173–180. 16508792 10.1007/s00114-006-0090-5

[102] Baeta R, Faivre B, Motreuil S, Gaillard M, Moreau J (2008) Carotenoid trade-off between parasitic resistance and sexual display: an experimental study in the blackbird (Turdus merula). Proc R Soc B 275: 427–434. 10.1098/rspb.2007.1383 PMC2596825 18055388

[103] Bédécarrats GY, Leeson S (2006) Dietary Lutein Influences Immune Response in Laying Hens. J Appl Poultry Res 15: 183–189.

[104] Benito MM, González-Solís J, Becker PH (2011) Carotenoid supplementation and sex-specific trade-offs between colouration and condition in common tern chicks. J Comp Physiol B 181: 539–549. 21153646 10.1007/s00360-010-0537-z

[105] Bertrand S, Alonso-Álvarez C, Devevey G, Faivre B, Prost J, et al (2006) Carotenoids modulate the trade-off between egg production and resistance to oxidative stress in zebra finches. Oecologia 147: 576–584. 16341888 10.1007/s00442-005-0317-8

[106] Biard C, Hardy C, Motreuil S, Moreau J (2009) Dynamics of PHA-induced immune response and plasma carotenoids in birds: should we have a closer look? J Exp Biol 212: 1336–1343. 19376954 10.1242/jeb.028449

[107] Biard C, Saulnier N, Gaillard M, Moreau J (2010) Carotenoid-based bill colour is an integrative signal of multiple parasite infection in blackbird. Naturwissenschaften 97: 987–995. 20845023 10.1007/s00114-010-0716-5

[108] Biard C, Surai PF, Møller AP (2007) An analysis of pre-and post-hatching maternal effects mediated by carotenoids in the blue tit. J Evol Biol 20: 326–339. 17210026 10.1111/j.1420-9101.2006.01194.x

[109] Biard C, Surai PF, Møller AP (2006) Carotenoid availability in diet and phenotype of blue and great tit nestlings. J Exp Biol 209: 1004–1015. 16513926 10.1242/jeb.02089

[110] Birkhead TR, Fletcher F, Pellatt EJ (1998) Sexual selection in the zebra finch Taeniopygia guttata: condition, sex traits and immune capacity. Behav Ecol Sociobiol 44: 179–191.

[111] Blas J, Pérez-Rodríguez L, Bortolotti GR, Viñuela J, Marchant TA (2006) Testosterone increases bioavailability of carotenoids: insights into the honesty of sexual signaling. P Natl Acad Sci U S A 103: 18633–18637. 10.1073/pnas.0609189103 PMC1660487 17121984

[112] Blount JD, Pike TW (2011) Deleterious effects of light exposure on immunity and sexual coloration in birds. Funct Ecol 26: 37–45.

[113] Blount JD, Surai PF, Nager RG, Houston DC, Møller AP, et al (2002) Carotenoids and egg quality in the lesser black-backed gull Larus fuscus: a supplemental feeding study of maternal effects. Proc R Soc B 269: 29–36. 10.1098/rspb.2001.1840 PMC1690857 11788033

[114] Bonisoli-Alquati A, Rubolini D, Caprioli M, Ambrosini R, Romano M, et al (2011) Egg testosterone affects wattle color and trait covariation in the ring-necked pheasant. Behav Ecol Sociobiol 65: 1779–1790.

[115] Bortolotti GR, Fernie KJ, Smits JE (2003) Carotenoid concentration and coloration of American Kestrels (Falco sparverius) disrupted by experimental exposure to PCBs. Funct Ecol 17: 651–657.

[116] Bortolotti GR, Negro JJ, Tella JL, Marchant TA, Bird DM (1996) Sexual dichromatism in birds independent of diet, parasites and androgens. Proc R Soc B 263: 1171–1176.

[117] Bortolotti GR, Tella JL, Forero MG, Dawson RD, Negro JJ (2000) Genetics, local environment and health as factors influencing plasma carotenoids in wild American kestrels (Falco sparverius). Proc R Soc B 267: 1433–1438. 10.1098/rspb.2000.1160 PMC1690695 10983827

[118] Bright A, Waas JR, King CM, Cuming PD (2004) Bill colour and correlates of male quality in blackbirds: an analysis using canonical ordination. Behav process 65: 123–132. 10.1016/j.beproc.2003.08.003 15222961

[119] Burley N, Tidemann SC, Halupka (1991) Bill colour and parasite levels of zebra finches. In: Loye JE, Zuk M, editors. Bird-parasite interactions. Oxford, UK: Oxford University Press. 359–376.

[120] Butler MWM, McGraw KJ (2011) Past or present? Relative contributions of developmental and adult conditions to adult immune function and coloration in mallard ducks (Anas platyrhynchos). J Comp Physiol B 181: 551–563. 21140156 10.1007/s00360-010-0529-z

[121] Butler MW, McGraw KJ (2010) Relationships between dietary carotenoids, body tissue carotenoids, parasite burden, and health state in wild mallard (Anas platyrhynchos) ducklings. Archiv Biochem Biophys 504: 154–160. 10.1016/j.abb.2010.07.003 20637173

[122] Camplani A, Saino N, Møller AP (1999) Carotenoids, sexual signals and immune function in barn swallows from Chernobyl. Proc R Soc B 266: 1111–1116. 10.1098/rspb.1999.0751 PMC1689949 10406129

[123] Casagrande S, Csermely D, Pini E (2006) Skin carotenoid concentration correlates with male hunting skill and territory quality in the kestrel Falco tinnunculus. J Avian Biol 37: 190–196.

[124] Casagrande S, Dell’Omo G, Costantini D, Tagliavini J, Groothuis T (2011) Variation of a carotenoid-based trait in relation to oxidative stress and endocrine status during the breeding season in the Eurasian kestrel: a multi-factorial study. Comp Biochem Phys A 160: 16–26. 10.1016/j.cbpa.2011.04.011 21620990

[125] Chui CKS, McGraw KJ, Doucet SM (2011) Carotenoid-based plumage coloration in golden-crowned kinglets Regulus satrapa: pigment characterization and relationships with migratory timing and condition. J Avian Biol 42: 309–322.

[126] Costantini D, Dell’Omo G (2006) Effects of T-cell-mediated immune response on avian oxidative stress. Comp Biochem Phys A 145: 137–142. 10.1016/j.cbpa.2006.06.002 16872854

[127] Costantini D, Coluzza C, Fanfani A, Dell’Omo G (2007) Effects of carotenoid supplementation on colour expression, oxidative stress and body mass in rehabilitated captive adult kestrels (Falco tinnunculus). J Comp Physiol B 177: 723–731. 17549495 10.1007/s00360-007-0169-0

[128] Costantini D, Fanfani A, Dell’Omo G (2007) Carotenoid availability does not limit the capability of nestling kestrels (Falco tinnunculus) to cope with oxidative stress. J Exp Biol 210: 1238–1244. 17371922 10.1242/jeb.002741

[129] Costantini D, Fanfani A, Dell’Omo G (2008) Effects of corticosteroids on oxidative damage and circulating carotenoids in captive adult kestrels (Falco tinnunculus). J Comp Physiol B 178: 829–835. 18443799 10.1007/s00360-008-0270-z

[130] Cote J, Arnoux E, Sorci G, Gaillard M, Faivre B (2010) Age-dependent allocation of carotenoids to coloration versus antioxidant defences. J Exp Biol 213: 271–277. 20038661 10.1242/jeb.035188

[131] Cucco M, Guasco B, Malacarne G, Ottonelli R (2006) Effects of β-carotene supplementation on chick growth, immune status and behaviour in the grey partridge, Perdix perdix. Behav process 73: 325–332. 10.1016/j.beproc.2006.08.002 16963199

[132] Cucco M, Guasco B, Malacarne G, Ottonelli R (2007) Effects of beta-carotene on adult immune condition and antibacterial activity in the eggs of the Grey Partridge, Perdix perdix. Comp Biochem Phys A 147: 1038–1046. 10.1016/j.cbpa.2007.03.014 17462926

[133] Dawson RD, Bortolotti GR (2006) Carotenoid-dependent coloration of male American kestrels predicts ability to reduce parasitic infections. Naturwissenschaften 93: 597–602. 16912887 10.1007/s00114-006-0146-6

[134] de Ayala RM, Saino N, Møller AP, Anselmi C (2007) Mouth coloration of nestlings covaries with offspring quality and influences parental feeding behavior. Behav Ecol 18: 526–534.

[135] del Cerro S, Merino S, Martínez-de la Puente J, Lobato E, Ruiz-de-Castañeda R, et al (2010) Carotenoid-based plumage colouration is associated with blood parasite richness and stress protein levels in blue tits (Cyanistes caeruleus). Oecologia 162: 825–835. 19937348 10.1007/s00442-009-1510-y

[136] Dufva R, Allander K (1995) Intraspecific Variation in Plumage Coloration Reflects Immune-Response in Great Tit (Parus-Major) Males. Funct Ecol 9: 785–789.

[137] Dugas MB, McGraw KJ (2011) Proximate Correlates of Carotenoid-Based Mouth Coloration in Nestling House Sparrows. The Condor 113: 691–700.

[138] Dunn PO, Garvin JC, Whittingham LA, Freeman-Gallant CR, Hasselquist D (2010) Carotenoid and melanin-based ornaments signal similar aspects of male quality in two populations of the common yellowthroat. Funct Ecol 24: 149–158.

[139] Edler AU, Friedl TWP (2010) Individual quality and carotenoid-based plumage ornaments in male red bishops (Euplectes orix): plumage is not all that counts. Biol J Linn Soc 99: 384–397.

[140] Eeva T, Sillanpää S, Salminen JP (2009) The effects of diet quality and quantity on plumage colour and growth of great tit Parus major nestlings: a food manipulation experiment along a pollution gradient. J Avian Biol 40: 491–499.

[141] Eraud C, Devevey G, Gaillard M, Prost J, Sorci G, et al (2007) Environmental stress affects the expression of a carotenoid-based sexual trait in male zebra finches. J Exp Biol 210: 3571–3578. 17921158 10.1242/jeb.005496

[142] Faivre B, Préault M, Salvadori F, Théry M, Gaillard M, et al (2003) Bill colour and immunocompetence in the European blackbird. Anim Behav 65: 1125–1131.

[143] Fenoglio S, Cucco M, Malacarne G (2002) Bill colour and body condition in the Moorhen Gallinula chloropus. Bird Study 49: 89–92.

[144] Fenoglio S, Cucco M, Malacarne G (2002) The effect of a carotenoid-rich diet on immunocompetence and behavioural performances in Moorhen chicks. Ethol Ecol Evol 14: 149–156.

[145] Fenoglio S, Cucco M, Fracchia L, Martinotti MG, Malacarne G (2004) Shield colours of the Moorhen are differently related to bacterial presence and health parameters. Ethol Ecol Evol 16: 171–180.

[146] Figuerola J, Domenech J, Senar JC (2003) Plumage colour is related to ectosymbiont load during moult in the serin, Serinus serinus: an experimental study. Anim Behav 65: 551–557.

[147] Figuerola J, Mũnoz E, Gutiérrez R, Ferrer D (1999) Blood parasites, leucocytes and plumage brightness in the Cirl Bunting, Emberiza cirlus. Funct Ecol 13: 594–601.

[148] Figuerola J, Torres J, Garrido J, Green AJ, Negro JJ (2005) Do carotenoids and spleen size vary with helminth load in greylag geese? Can J Zool 83: 389–395.

[149] Fitze PS, Tschirren B, Gasparini J, Richner H (2007) Carotenoid-based plumage colors and immune function: is there a trade-off for rare carotenoids? Am Nat 169 Suppl 1S137–S144. 19426088 10.1086/510094

[150] Freeman-Gallant CR, Amidon J, Berdy B, Wein S, Taff CC, et al (2011) Oxidative damage to DNA related to survivorship and carotenoid-based sexual ornamentation in the common yellowthroat. Biol Lett 7: 429–432. 21247942 10.1098/rsbl.2010.1186 PMC3097884

[151] Galván I, Diaz L, Jose Sanz J (2009) Relationships between territory quality and carotenoid-based plumage colour, cell-mediated immune response, and body mass in Great Tit Parus major nestlings. Acta Ornithol 44: 139–150.

[152] Garvin JC, Dunn PO, Whittingham LA, Steeber DA, Hasselquist D (2008) Do male ornaments signal immunity in the common yellowthroat? Behav Ecol 19: 54–60.

[153] Gladbach A, Gladbach DJ, Kempenaers B, Quillfeldt P (2010) Female-specific colouration, carotenoids and reproductive investment in a dichromatic species, the upland goose Chloephaga picta leucoptera. Behav Ecol Sociobiol 64: 1779–1789. 20976290 10.1007/s00265-010-0990-4 PMC2952766

[154] Gladbach A, Gladbach DJ, Quillfeldt P (2010) Variations in leucocyte profiles and plasma biochemistry are related to different aspects of parental investment in male and female Upland geese Chloephaga picta leucoptera. Comp Biochem Phys A 156: 269–277. 10.1016/j.cbpa.2010.02.012 20176125

[155] Hadfield JD, Owens IPF (2006) Strong environmental determination of a carotenoid-based plumage trait is not mediated by carotenoid availability. J Evol Biol 19: 1104–1114. 16780511 10.1111/j.1420-9101.2006.01095.x

[156] Häsä L (2006) Health parameters and sexual signalling in yearling black grouse males (Tetrao tetrix). Msc thesis, University of Jyväskylä.

[157] Hill GE, Hood WR, Huggins K (2009) A multifactorial test of the effects of carotenoid access, food intake and parasite load on the production of ornamental feathers and bill coloration in American goldfinches. J Exp Biol 212: 1225–1233. 19329755 10.1242/jeb.026963

[158] Hill GE, Montgomerie R, Inouye CY, Dale J (1994) Influence of dietary carotenoids on plasma and plumage colour in the house finch: intra-and intersexual variation. Funct Ecol 8: 343–350.

[159] Saks L, Ots I, Hõrak P (2003) Carotenoid-based plumage coloration of male greenfinches reflects health and immunocompetence. Oecologia 134: 301–307. 12647136 10.1007/s00442-002-1125-z

[160] Hõrak P, Saks L, Karu U, Ots I, Surai PF, et al (2004) How coccidian parasites affect health and appearance of greenfinches. J Anim Ecol 73: 935–947.

[161] Hõrak P, Saks L, Zilmer M, Karu U, Zilmer K (2007) Do dietary antioxidants alleviate the cost of immune activation? An experiment with greenfinches. Am Nat 170: 625–635. 17891740 10.1086/521232

[162] Hõrak P, Sild E, Soomets U, Sepp T, Kilk K (2010) Oxidative stress and information content of black and yellow plumage coloration: an experiment with greenfinches. J Exp Biol 213: 2225–2233. 20543121 10.1242/jeb.042085

[163] Hõrak P, Surai PF, Ots I, Møller AP (2004) Fat soluble antioxidants in brood-rearing great tits Parus major: relations to health and appearance. J Avian Biol 35: 63–70.

[164] Hõrak P, Zilmer M, Saks L, Ots I, Karu U, et al (2006) Antioxidant protection, carotenoids and the costs of immune challenge in greenfinches. J Exp Biol 209: 4329–4338. 17050848 10.1242/jeb.02502

[165] Isaksson C, McLaughlin P, Monaghan P, Andersson S (2007) Carotenoid pigmentation does not reflect total non-enzymatic antioxidant activity in plasma of adult and nestling great tits, Parus major. Funct Ecol 21: 1123–1129.

[166] Jouventin P, McGraw KJ, Morel M, Célerier A (2007) Dietary carotenoid supplementation affects orange beak but not foot coloration in gentoo penguins Pygoscelis papua. Waterbirds 30: 573–578.

[167] Karu U, Saks L, Hõrak P (2007) Carotenoid coloration in greenfinches is individually consistent irrespective of foraging ability. Physiol Biochem Zool 80: 663–670. 17910002 10.1086/521084

[168] Larcombe SD, Mullen W, Alexander L, Arnold KE (2010) Dietary antioxidants, lipid peroxidation and plumage colouration in nestling blue tits Cyanistes caeruleus. Naturwissenschaften 97: 903–913. 20838757 10.1007/s00114-010-0708-5

[169] Larcombe S, Tregaskes C, Coffey J, Stevenson A, Alexander L, et al (2008) The effects of short-term antioxidant supplementation on oxidative stress and flight performance in adult budgerigars Melopsittacus undulatus. J Exp Biol 211: 2859–2864. 18723545 10.1242/jeb.017970

[170] Leclaire S, White J, Arnoux E, Faivre B, Vetter N, et al (2011) Integument coloration signals reproductive success, heterozygosity, and antioxidant levels in chick-rearing black-legged kittiwakes. Naturwissenschaften 98: 773–782. 21792598 10.1007/s00114-011-0827-7

[171] Losdat S, Helfenstein F, Gaude B, Richner H (2011) Reproductive effort transiently reduces antioxidant capacity in a wild bird. Behav Ecol 22: 1218–1226.

[172] Losdat S, Richner H, Blount JD, Helfenstein F (2011) Immune Activation Reduces Sperm Quality in the Great Tit. PLoS ONE 6: e22221. 21765955 10.1371/journal.pone.0022221 PMC3134482

[173] López G, Soriguer R, Figuerola J (2011) Is bill colouration in wild male Blackbirds (Turdus merula) related to biochemistry parameters and parasitism? J Ornithol 152: 965–973.

[174] Maney DL, Davis AK, Goode CT, Reid A, Showalter C (2008) Carotenoid-based plumage coloration predicts leukocyte parameters during the breeding season in northern cardinals (Cardinalis cardinalis). Ethology 114: 369–380.

[175] Martinez-Haro M, Green AJ, Mateo R (2011) Effects of lead exposure on oxidative stress biomarkers and plasma biochemistry in waterbirds in the field. Environ Res 111: 530–538. 21411076 10.1016/j.envres.2011.02.012

[176] Martínez-Padilla J, Mougeot F, Pérez-Rodríguez L, Bortolotti GR (2007) Nematode parasites reduce carotenoid-based signalling in male red grouse. Biol Lett 3: 161–164. 17264052 10.1098/rsbl.2006.0593 PMC2375928

[177] McGraw KJ, Ardia DR (2005) Sex differences in carotenoid status and immune performance in zebra finches. Evol Ecol Res 7: 251–262.

[178] McGraw KJ, Parker RS (2006) A novel lipoprotein-mediated mechanism controlling sexual attractiveness in a colorful songbird. Physiol Behav 87: 103–108. 16202433 10.1016/j.physbeh.2005.09.001

[179] McGraw KJ, Crino OL, Jerez WM (2006) Effect of dietary carotenoid supplementation on food intake and immune function in a songbird with no carotenoid coloration. Ethology 112: 1209–1216.

[180] McGraw KJ, Gregory AJ, Parker RS, Adkins-Regan E (2003) Diet, plasma carotenoids, and sexual coloration in the zebra finch (Taeniopygia guttata). The Auk 120: 400–410.

[181] McGraw KJ, Nolan PM, Crino OL (2011) Carotenoids bolster immunity during moult in a wild songbird with sexually selected plumage coloration. Biol J Linn Soc 102: 560–572.

[182] McGraw KJ, Nolan PM, Crino OL (2006) Carotenoid accumulation strategies for becoming a colourful House Finch: analyses of plasma and liver pigments in wild moulting birds. Funct Ecol 20: 678–688.

[183] Merila J, Sheldon BC, Lindström K (1999) Plumage brightness in relation to haematozoan infections in the greenfinch Carduelis chloris: Bright males are a good bet. Ecoscience 6: 12–18.

[184] Morales J, Velando A, Torres R (2009) Fecundity compromises attractiveness when pigments are scarce. Behav Ecol 20: 117–123.

[185] Mougeot F, Pérez-Rodríguez L, Martinez-Padilla J, Leckie F, Redpath SM (2007) Parasites, testosterone and honest carotenoid-based signalling of health. Funct Ecol 21: 886–898.

[186] Mougeot F, Martinez-Padilla J, Blount JD, Pérez-Rodríguez L, Webster LMI, et al (2010) Oxidative stress and the effect of parasites on a carotenoid-based ornament. J Exp Biol 213: 400–407. 20086124 10.1242/jeb.037101

[187] Mougeot F, Pérez-Rodríguez L, Sumozas N, Terraube J (2009) Parasites, condition, immune responsiveness and carotenoid-based ornamentation in male red-legged partridge Alectoris rufa. J Avian Biol 40: 67–74.

[188] Navara KJ, Hill GE (2003) Dietary carotenoid pigments and immune function in a songbird with extensive carotenoid-based plumage coloration. Behav Ecol 14: 909–916.

[189] O’Brien EL, Dawson RD (2009) Palatability of passerines to parasites: within-brood variation in nestling responses to experimental parasite removal and carotenoid supplementation. Oikos 118: 1743–1751.

[190] Ohlsson T, Smith HG, Råberg L, Hasselquist D (2002) Pheasant sexual ornaments reflect nutritional conditions during early growth. Proc R Soc B 269: 21–27. 10.1098/rspb.2001.1848 PMC1690866 11788032

[191] Orledge JM, Blount JD, Hoodless AN, Pike TW, Royle NJ (2011) Synergistic effects of supplementation of dietary antioxidants during growth on adult phenotype in ring-necked pheasants, Phasianus colchicus. Funct Ecol 26: 254–264.

[192] Pap PL, Vágási CI, Czirjak GA, Titilincu A, Pintea A, et al (2011) The Effect of Coccidians on the Condition and Immune Profile of Molting House Sparrows (Passer domesticus). The Auk 128: 330–339.

[193] Pap PL (2002) Breeding time and sex-specific health status in the barn swallow (Hirundo rustica). Can J Zool 80: 2090–2099.

[194] Peluc SI, Reed WL, McGraw KJ, Gibbs P (2012) Carotenoid supplementation and GnRH challenges influence female endocrine physiology, immune function, and egg-yolk characteristics in Japanese quail (Coturnix japonica). J Comp Physiol B. In press. 10.1007/s00360-011-0638-3 22237302

[195] Pérez-Rodríguez L, Mougeot F, Alonso-Álvarez C (2010) Carotenoid-based coloration predicts resistance to oxidative damage during immune challenge. J Exp Biol 213: 1685–1690. 20435819 10.1242/jeb.039982

[196] Pérez-Rodríguez L, Mougeot F, Alonso-Álvarez C, Blas J, Viñuela J, et al (2008) Cell-mediated immune activation rapidly decreases plasma carotenoids but does not affect oxidative stress in red-legged partridges (Alectoris rufa). J Exp Biol 211: 2155–2161. 18552305 10.1242/jeb.017178

[197] Peters A, Delhey K, Andersson S, Van Noordwijk H, Förschler MI (2008) Condition-dependence of multiple carotenoid-based plumage traits: an experimental study. Funct Ecol 22: 831–839.

[198] Peters A, Delhey K, Johnsen A, Kempenaers B (2007) The condition-dependent development of carotenoid-based and structural plumage in nestling blue tits: males and females differ. Am Nat 169: S122–S136. 29517928 10.1086/510139

[199] Peters A, Denk AG, Delhey K, Kempenaers B (2004) Carotenoid-based bill colour as an indicator of immunocompetence and sperm performance in male mallards. J Evol Biol 17: 1111–1120. 15312083 10.1111/j.1420-9101.2004.00743.x

[200] Peters A, Magdeburg S, Delhey K (2011) The carotenoid conundrum: improved nutrition boosts plasma carotenoid levels but not immune benefits of carotenoid supplementation. Oecologia 166: 35–43. 21301878 10.1007/s00442-011-1921-4

[201] Pérez C, Lores M, Velando A (2010) Oil pollution increases plasma antioxidants but reduces coloration in a seabird. Oecologia 163: 875–884. 20532916 10.1007/s00442-010-1677-2

[202] Pérez-Rodríguez L (2007) Carotenoid-based ornamentation as a dynamic but consistent individual trait. Behav Ecol Sociobiol 62: 995–1005.

[203] Pérez-Rodríguez L, Viñuela J (2008) Carotenoid-based bill and eye ring coloration as honest signals of condition: an experimental test in the red-legged partridge (Alectoris rufa). Naturwissenschaften 95: 821–830. 18470503 10.1007/s00114-008-0389-5

[204] Quillfeldt P, Masello JF, Möstl E (2004) Blood chemistry in relation to nutrition and ectoparasite load in Wilson’s storm-petrels Oceanites oceanicus. Polar Biol 27: 168–176.

[205] Safran RJ, McGraw KJ, Wilkins MR, Hubbard JK, Marling J, et al (2010) Positive Carotenoid Balance Correlates with Greater Reproductive Performance in a Wild Bird. PLoS ONE 5: 823–827. 10.1371/journal.pone.0009420 PMC2828481 20195540

[206] Saino N, Ambrosini R, Martinelli R, Ninni P, Møller AP (2003) Gape coloration reliably reflects immunocompetence of barn swallow (Hirundo rustica) nestlings. Behav Ecol 14: 16–22.

[207] Saino N, Stradi R, Ninni P, Pini E, Møller AP (1999) Carotenoid plasma concentration, immune profile, and plumage ornamentation of male barn swallows (Hirundo rustica). Am Nat 154: 441–448. 10523490 10.1086/303246

[208] Sepp T, Karu U, Sild E, Männiste M, Hõrak P (2011) Effects of carotenoids, immune activation and immune suppression on the intensity of chronic coccidiosis in greenfinches. Exp Parasitol 127: 651–657. 21176774 10.1016/j.exppara.2010.12.004

[209] Seutin G (1994) Plumage redness in redpoll finches does not reflect hemoparasitic infection. Oikos 70: 280–286.

[210] Shanmugasundaram R, Selvaraj RK (2011) Lutein supplementation alters inflammatory cytokine production and antioxidant status in F-line turkeys. Poult Sci 90: 971–976. 21489941 10.3382/ps.2010-01150

[211] Sild E, Sepp T, Männiste M, Hõrak P (2011) Carotenoid intake does not affect immune-stimulated oxidative burst in greenfinches. J Exp Biol 214: 3467–3473. 21957110 10.1242/jeb.062182

[212] Smith CL, Toomey M, Walker BR, Braun EJ, Wolf BO, et al (2011) Naturally high plasma glucose levels in mourning doves (Zenaida macroura) do not lead to high levels of reactive oxygen species in the vasculature. Zoology 114: 171–176. 21600747 10.1016/j.zool.2010.12.001 PMC3766343

[213] Smith HG, Råberg L, Ohlsson T, Granbom M, Hasselquist D (2007) Carotenoid and protein supplementation have differential effects on pheasant ornamentation and immunity. J Evol Biol 20: 310–319. 17210024 10.1111/j.1420-9101.2006.01203.x

[214] Sternalski A, Mougeot F, Bretagnolle V (2012) Carotenoid limitation and allocation priorities in asynchronous raptor nestlings. Biol J Linn Soc 105: 13–24.

[215] Stirnemann I, Johnston G, Rich B, Robertson J, Kleindorfer S (2009) Phytohaemagglutinin (PHA) response and bill-hue wavelength increase with carotenoid supplementation in Diamond Firetails (Stagonopleura guttata). Emu 109: 344–351.

[216] Sundberg J (1995) Parasites, plumage coloration and reproductive success in the yellowhammer, Emberiza citrinella. Oikos 74: 331–339.

[217] Tengerdy RP, Lacetera NG, Nockels CF (1990) Effect of beta carotene on disease protection and humoral immunity in chickens. Avian Dis 34: 848–854. 2282015

[218] Thompson CW, Hillgarth N, Leu M, McClure HE (1997) High parasite load in house finches (Carpodacus mexicanus) is correlated with reduced expression of a sexually selected trait. Am Nat 149: 270–294.

[219] Thorogood R, Kilner RM, Karadaş F, Ewen JG (2008) Spectral mouth colour of nestlings changes with carotenoid availability. Funct Ecol 22: 1044–1051.

[220] Toomey MB, Butler MW, McGraw KJ (2010) Immune-system activation depletes retinal carotenoids in house finches (Carpodacus mexicanus). J Exp Biol 213: 1709–1716. 20435822 10.1242/jeb.041004

[221] Torres R, Velando A (2007) Male reproductive senescence: the price of immune-induced oxidative damage on sexual attractiveness in the blue-footed booby. J Anim Ecol 76: 1161–1168. 17922712 10.1111/j.1365-2656.2007.01282.x

[222] Travers M (2009) Nest predation, clutch size, and physiological costs of egg production in the song sparrow *(Melospiza melodia)*. Bsc thesis, Bishop’s University.

[223] Tschirren B, Fitze PS, Richner H (2003) Proximate mechanisms of variation in the carotenoid-based plumage coloration of nestling great tits (Parus major L.). J Evol Biol 16: 91–100. 14635884 10.1046/j.1420-9101.2003.00483.x

[224] Tummeleht L, Mägi M, Kilgas P, Mänd R, Hõrak P (2006) Antioxidant protection and plasma carotenoids of incubating great tits (Parus major L.) in relation to health state and breeding conditions. Comp Biochem Phys C 144: 166–172. 10.1016/j.cbpc.2006.08.004 17035099

[225] Vinkler M, Schnitzer J, Munclinger P, Albrecht T (2012) Phytohaemagglutinin skin-swelling test in scarlet rosefinch males: low-quality birds respond more strongly. Anim Behav 83: 17–23.

[226] Weatherhead PJ, Metz KJ, Bennett GF, Irwin RE (1993) Parasite Faunas, Testosterone and Secondary Sexual Traits in Male Red-Winged Blackbirds. Behav Ecol Sociobiol 33: 13–23.

[227] Wiehn J, Korpimäki E, Bildstein K, Sorjonen J (1997) Mate choice and reproductive success in the American kestrel: A role for blood parasites? Ethology 103: 304–317.

[228] Woodall AA, Britton G, Jackson MJ (1996) Dietary supplementation with carotenoids: effects on α-tocopherol levels and susceptibility of tissues to oxidative stress. Br J Nutr 76: 307–317. 8813904 10.1079/bjn19960034

[229] Zhang W, Zhang KY, Ding XM, Bai S, Hernandez JM, et al (2011) Influence of canthaxanthin on broiler breeder reproduction, chick quality, and performance. Poult Sci 90: 1516–1522. 21673167 10.3382/ps.2010-01126

[230] Isaksson C, Andersson S (2008) Oxidative stress does not influence carotenoid mobilization and plumage pigmentation. Proc R Soc B 275: 309–314. 10.1098/rspb.2007.1474 PMC2593728 18029305

[231] Galván I, Alonso-Álvarez C (2009) The expression of melanin-based plumage is separately modulated by exogenous oxidative stress and a melanocortin. Proc R Soc B 276: 3089–3097. 10.1098/rspb.2009.0774 PMC2817136 19520801

[232] Saino N, Caprioli M, Romano M, Boncoraglio G, Rubolini D, et al (2011) Antioxidant Defenses Predict Long-Term Survival in a Passerine Bird. PLoS ONE 6: e19593. 21573124 10.1371/journal.pone.0019593 PMC3089629

[233] Hõrak P, Cohen A (2010) How to measure oxidative stress in an ecological context: methodological and statistical issues. Funct Ecol 24: 960–970.

[234] Hill GE (2006) Female mate choice for ornamental coloration. In: Hill GE, McGraw KJ, editors. Bird coloration: Function and Evolution. Cambridge: Harvard University press. 137–200.

[235] Bell AM, Hankison SJ, Laskowski KL (2009) The repeatability of behaviour: a meta-analysis. Anim Behav 77: 771–783. 24707058 10.1016/j.anbehav.2008.12.022 PMC3972767

[236] Blount JD, McGraw KJ (2008) The signal functions of carotenoid colouration in plants and animals. In: Britton G, Liaaen-Jensen S, Pfander H, editors. Carotenoids, volume 4: natural functions. Basel, Switzerland: Birkhauser. 213–236.

