# Supplementary figures and images for: What Does Carotenoid-Dependent Coloration Tell? Plasma Carotenoid Level Signals Immunocompetence and Oxidative Stress State in Birds–A Meta-Analysis
Source: PLoS One. 2012 Aug 14;7(8):e43088. doi: 10.1371/journal.pone.0043088 (PMC3419220; doi:10.1371/journal.pone.0043088)

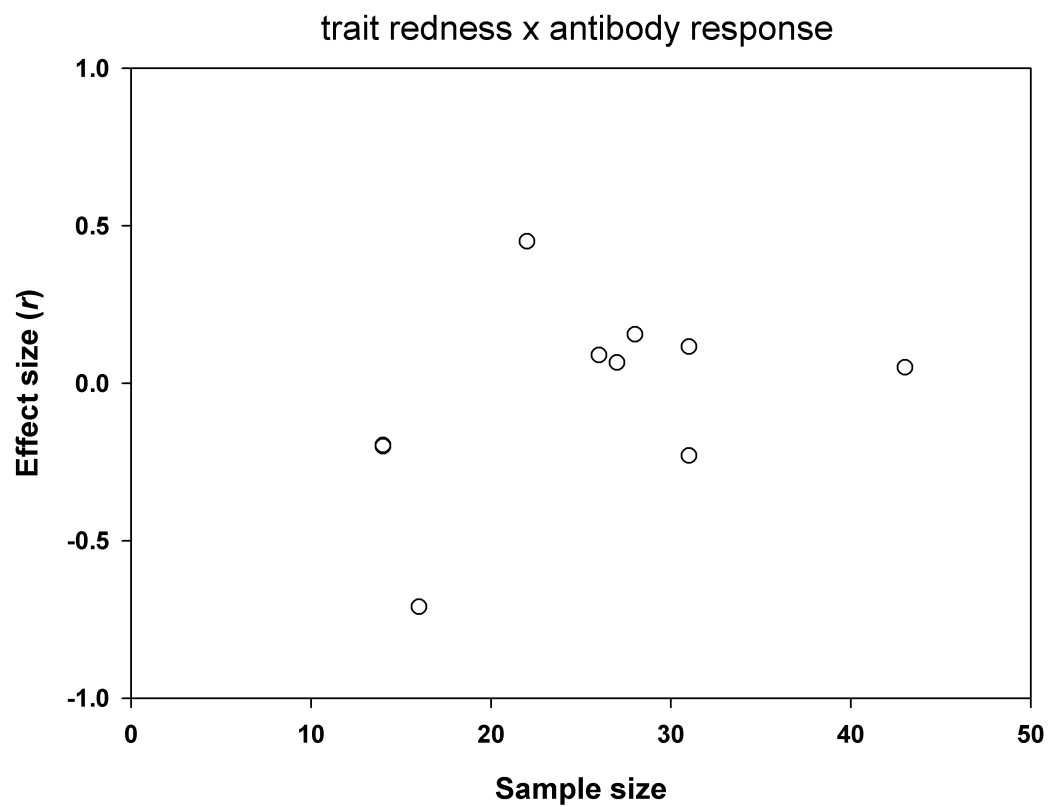

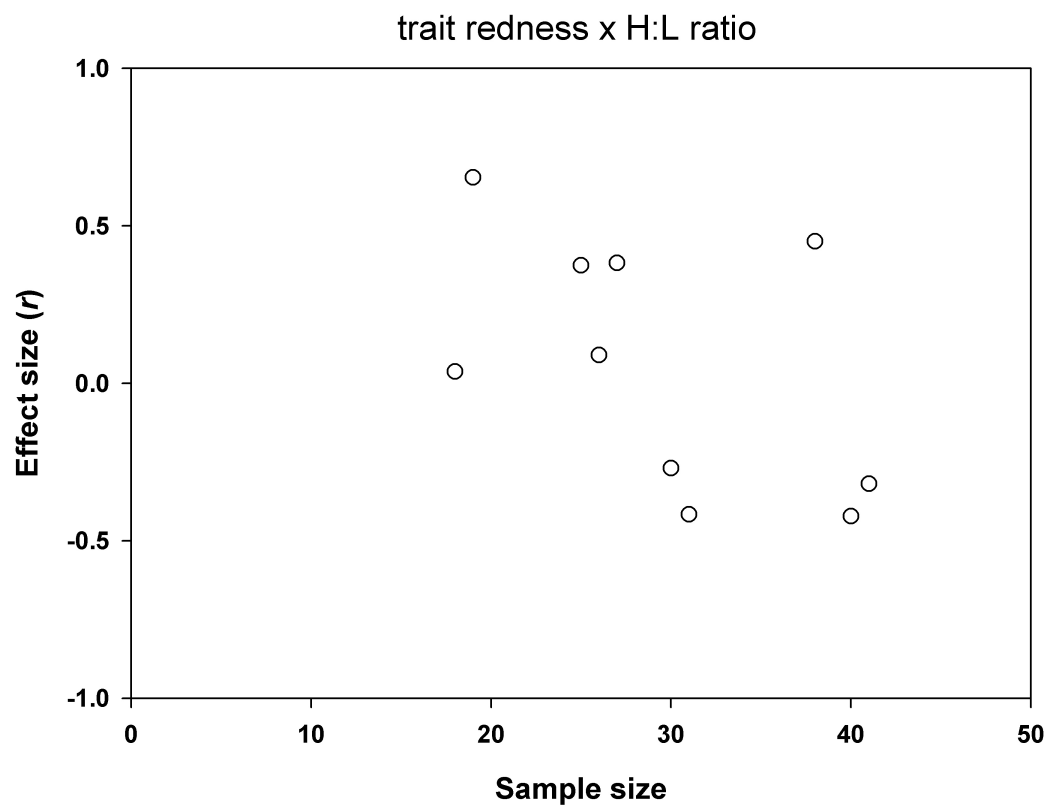

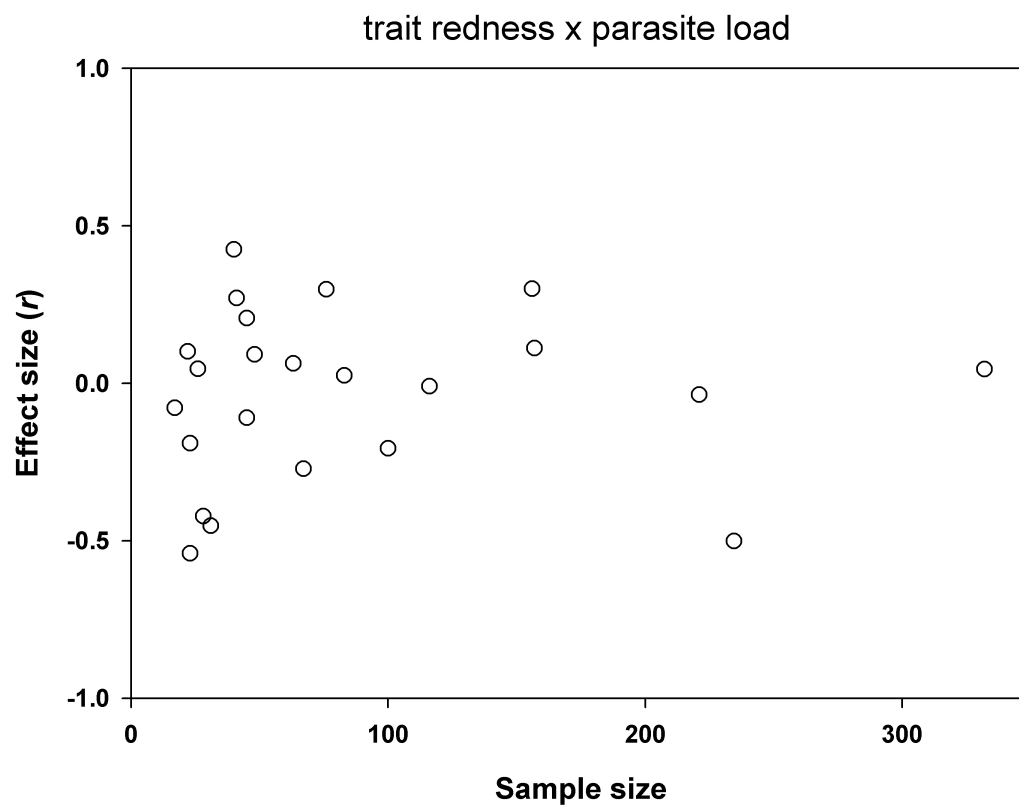

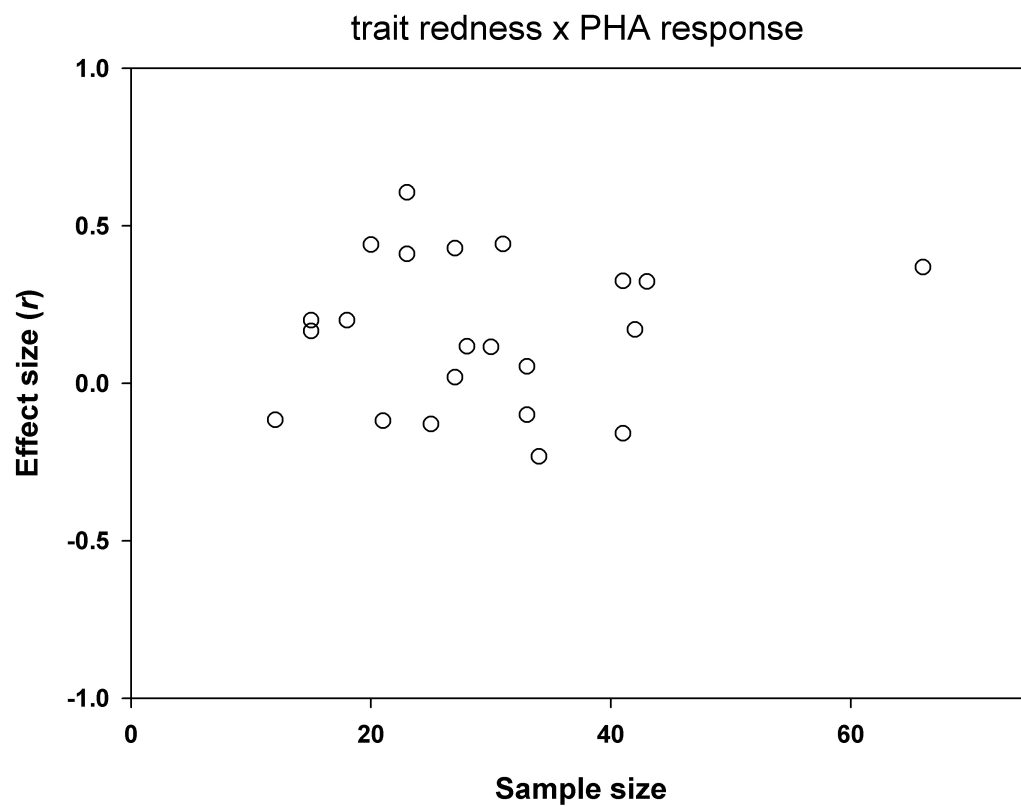

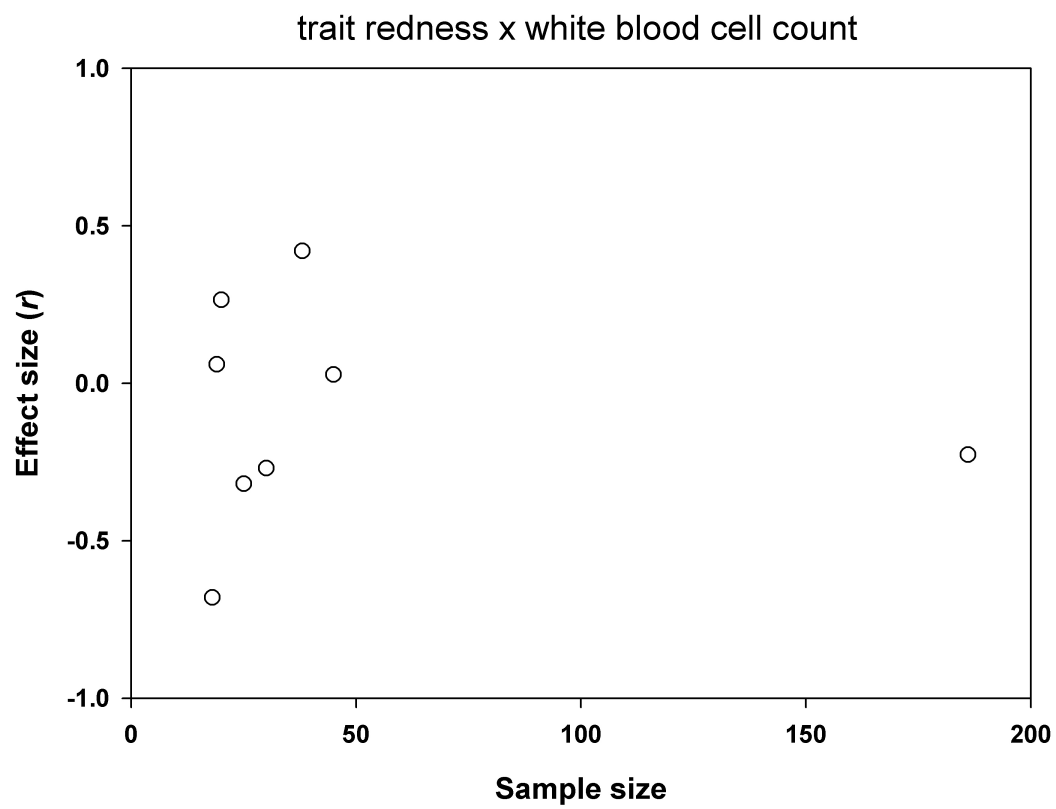

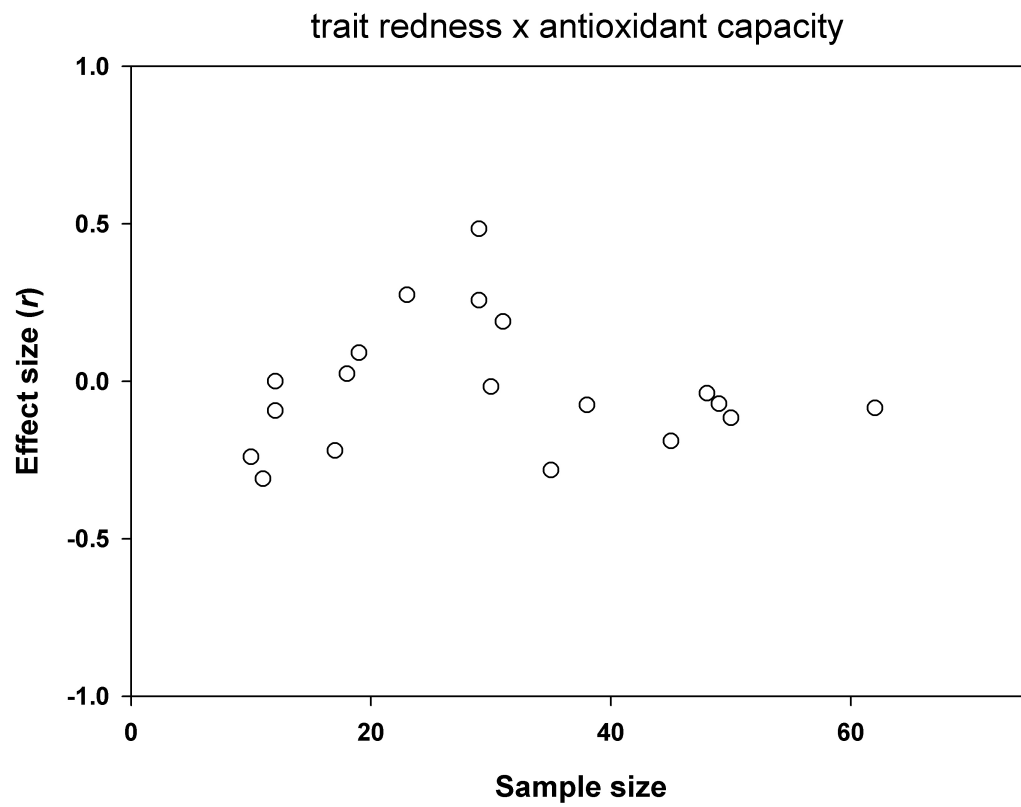

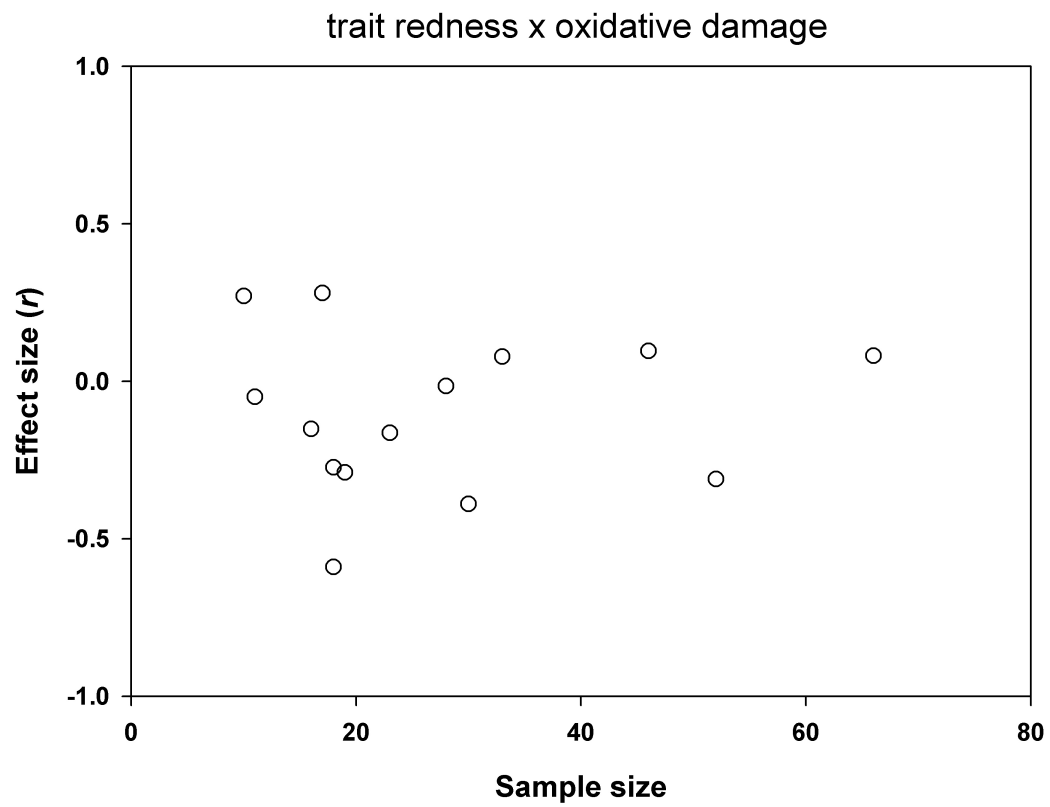

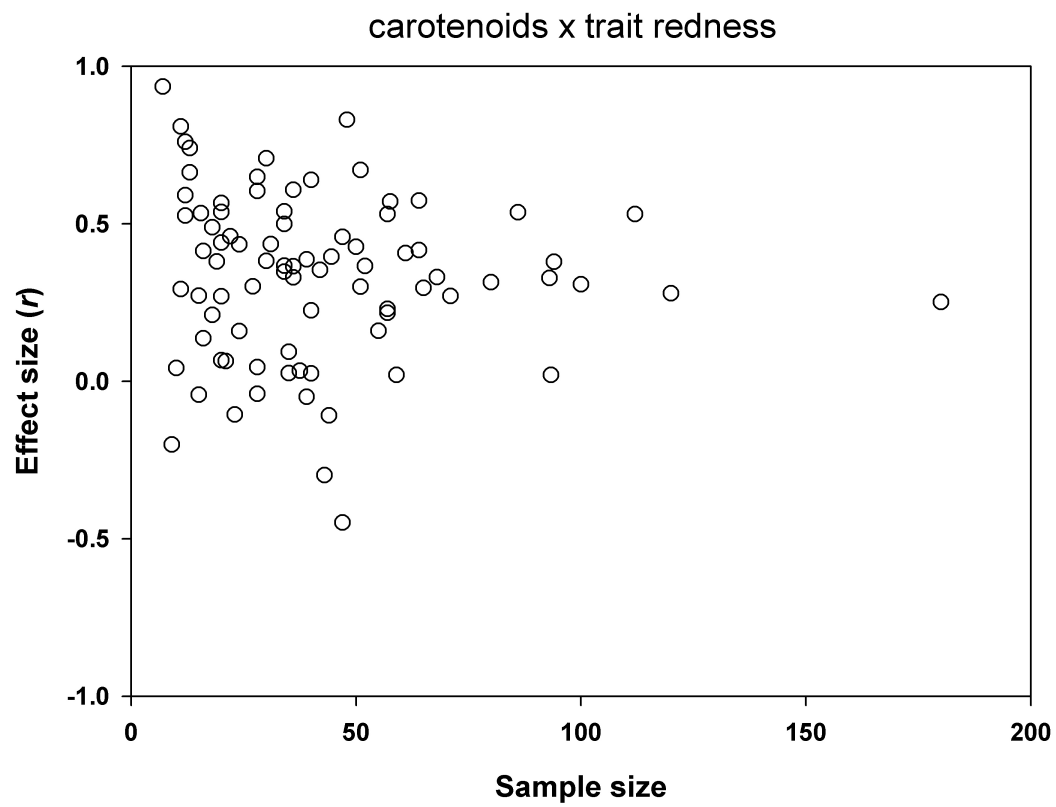

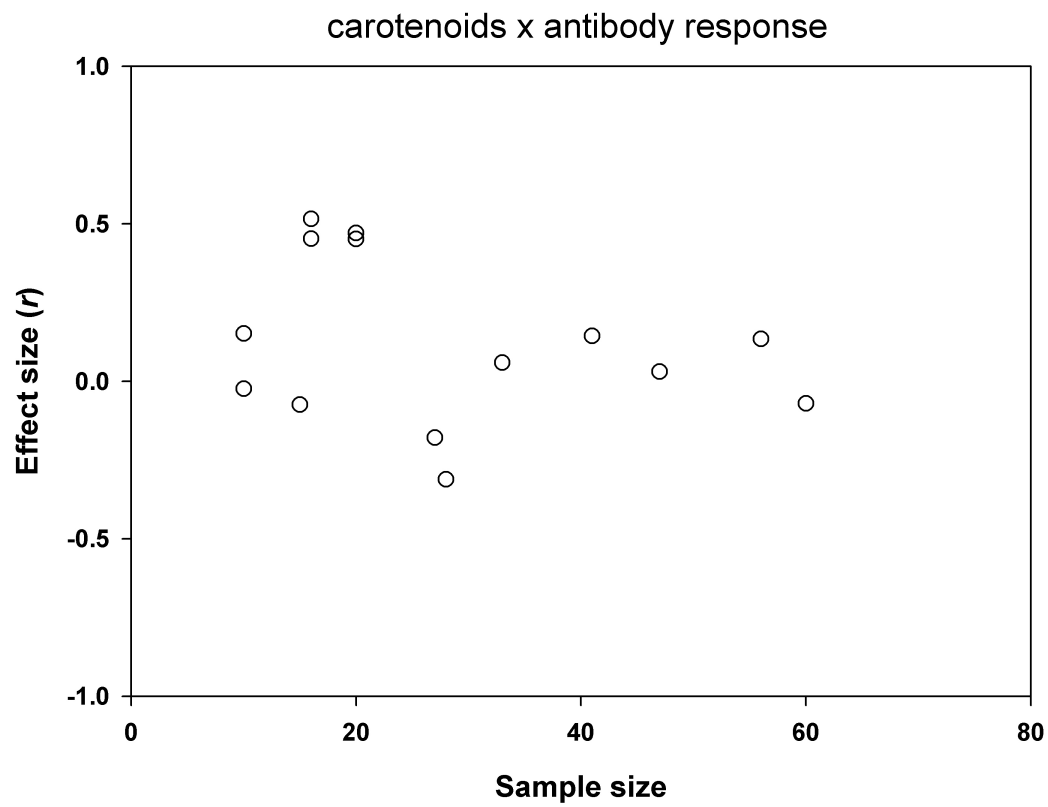

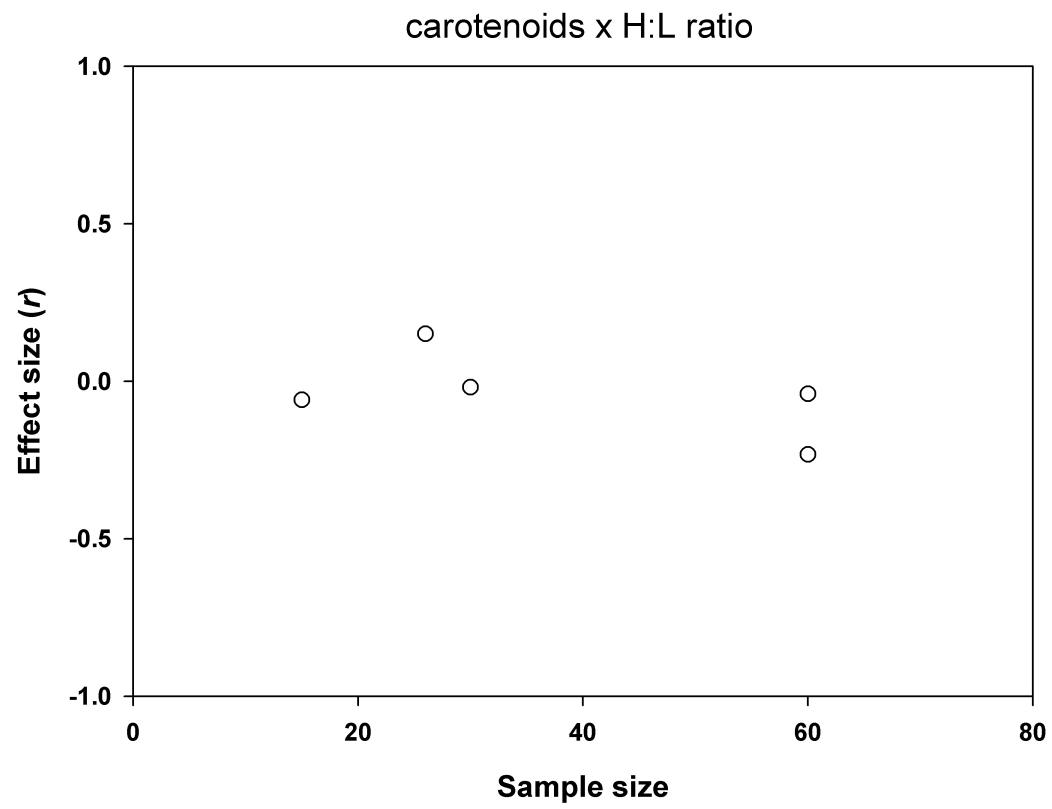

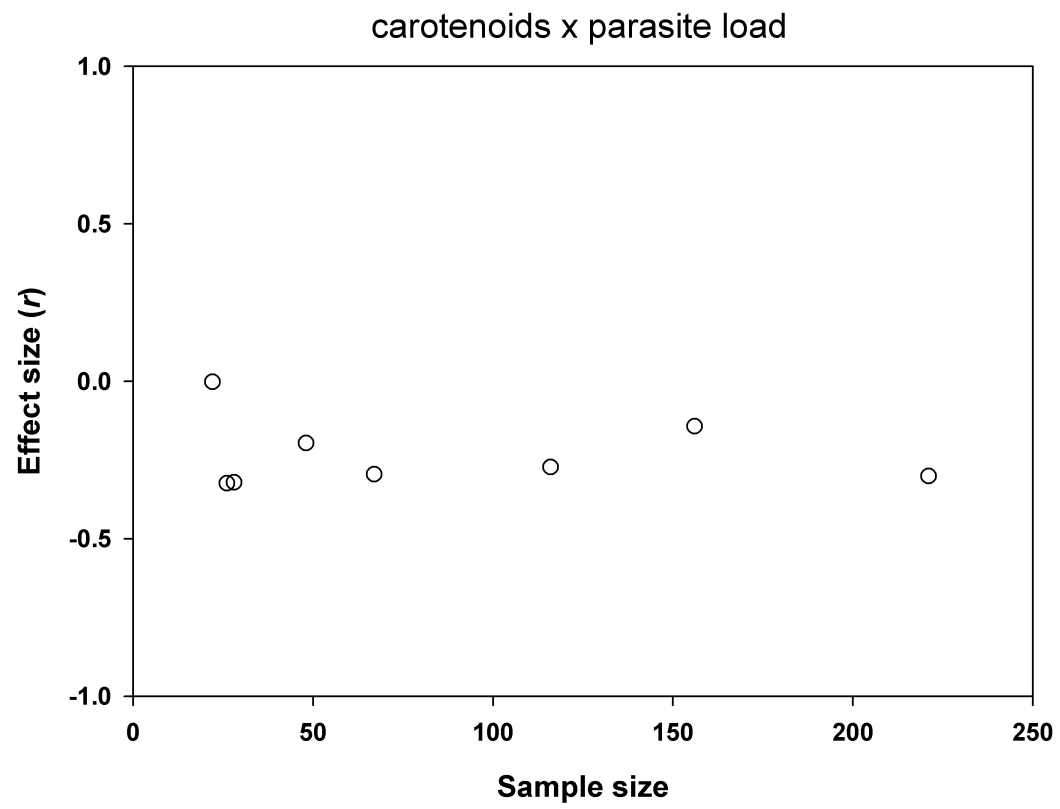

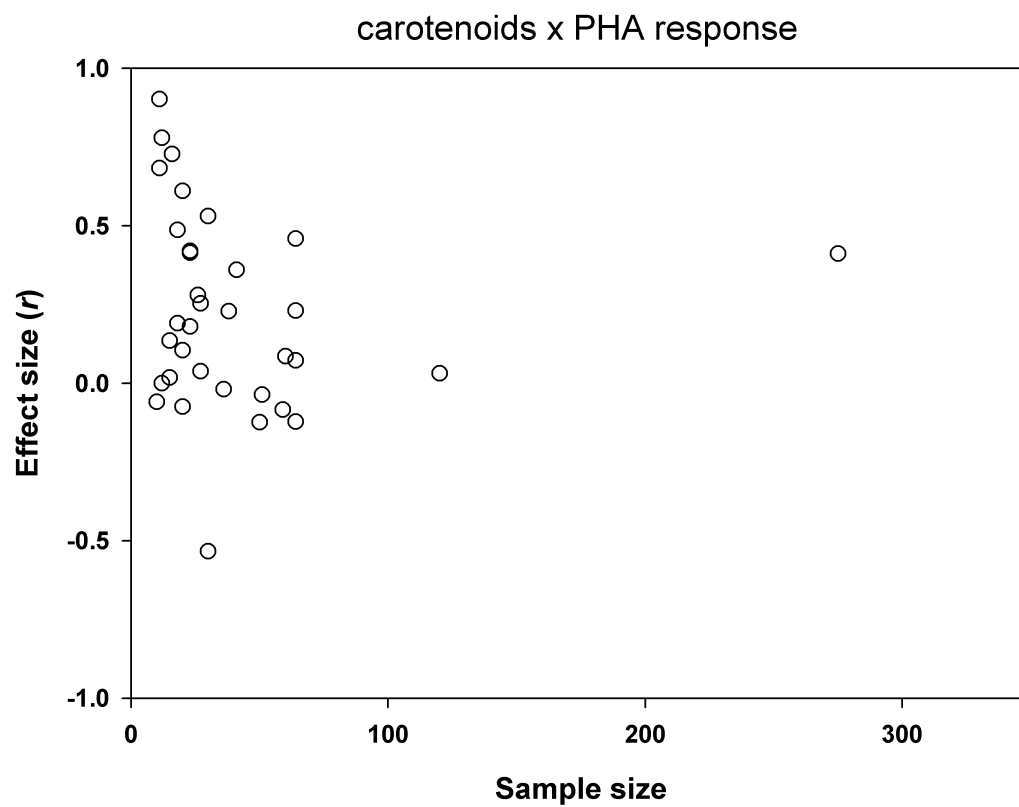

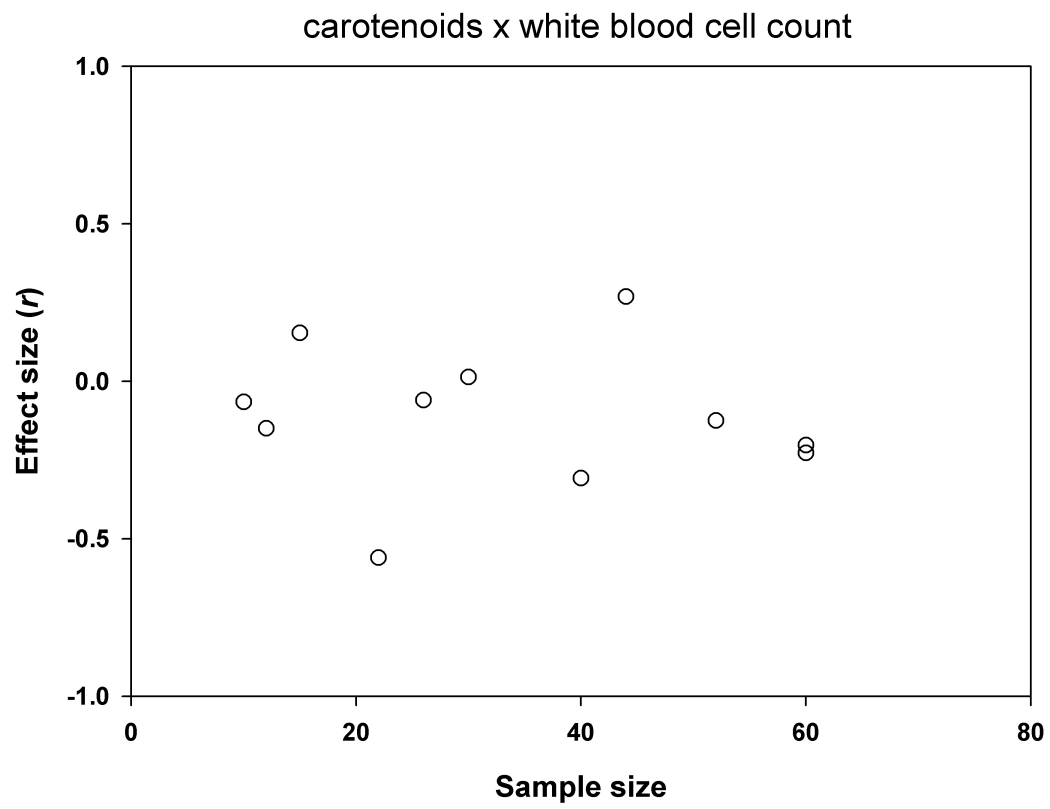

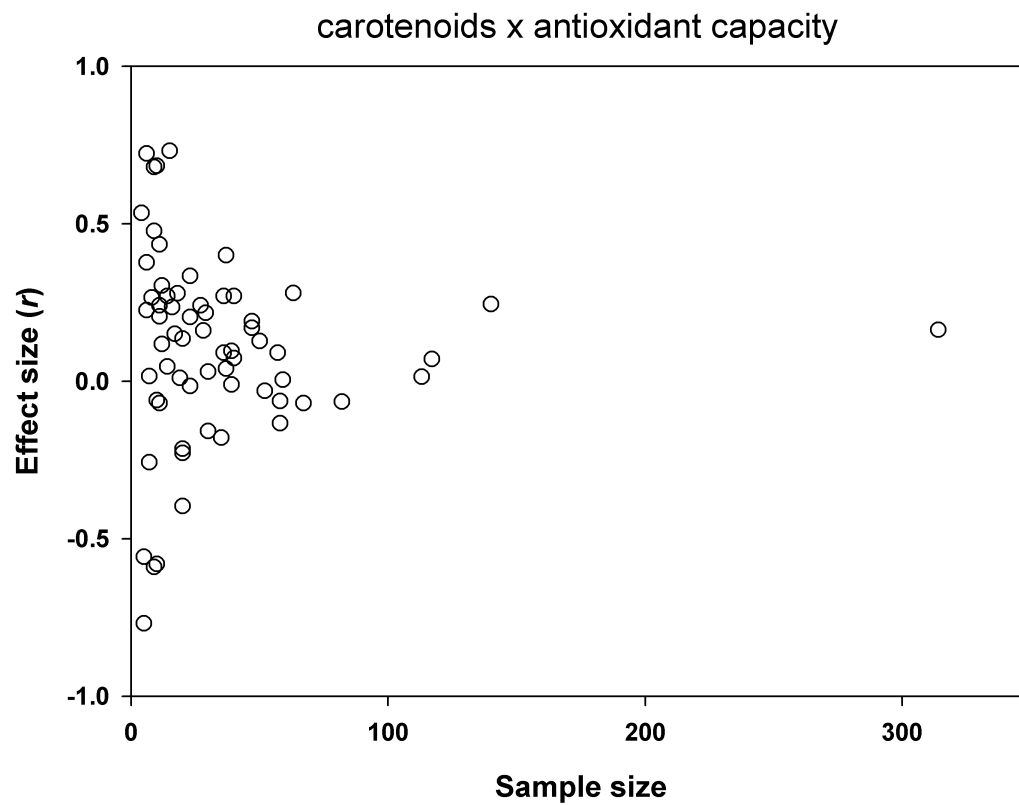

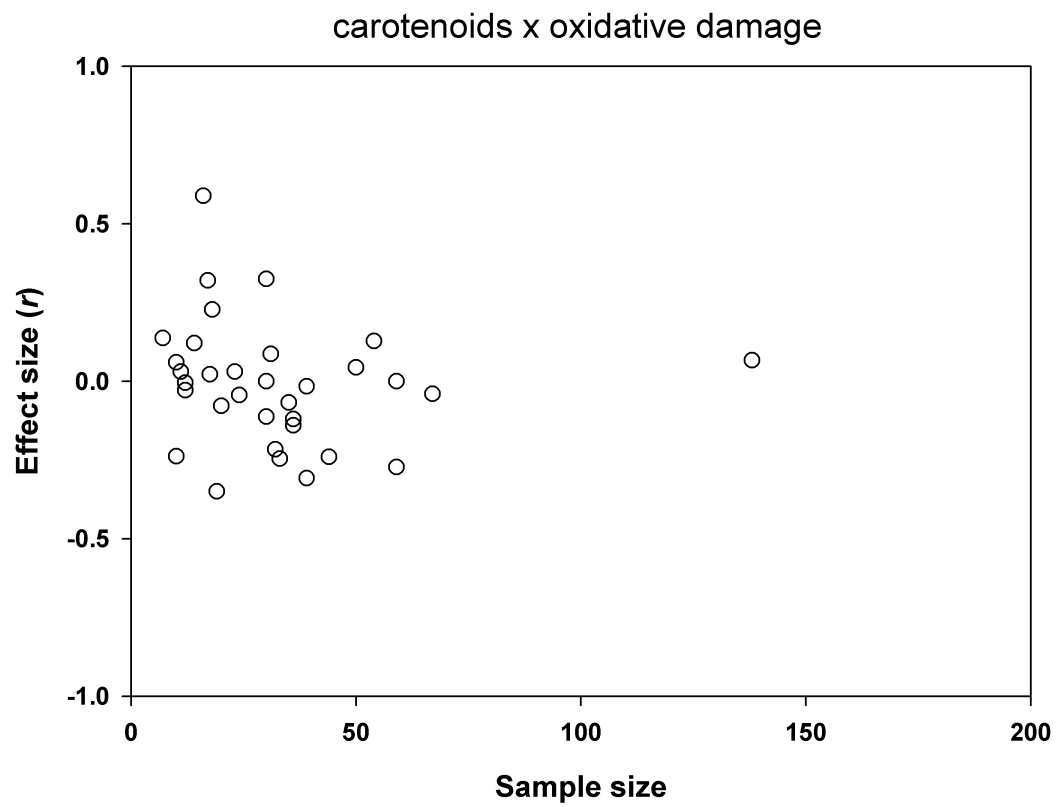

Supplement: Funnel Plots S1 — Plotted in the order of table 1 and data S1 are the separate datasets of effect sizes plotted against their corresponding sample sizes. The title depicts the relationship plotted. (PDF) [file pone.0043088.s001.pdf]
