# Supplementary material for: What Does Carotenoid-Dependent Coloration Tell? Plasma Carotenoid Level Signals Immunocompetence and Oxidative Stress State in Birds–A Meta-Analysis
Source: PLoS One. 2012 Aug 14;7(8):e43088. doi: 10.1371/journal.pone.0043088 (PMC3419220; doi:10.1371/journal.pone.0043088)
Supplement: PRISMA S1 — PRISMA flow diagram showing the literature search procedure. (PDF) [file pone.0043088.s003.pdf]

PRISMA 2009 Flow Diagram

Title corresponding manuscript: Higher carotenoid availability signals higher immunocompetence and lowered oxidative stress state in birds – a meta-analysis

Search terms in Google Scholar:
("immune function" OR immunocompetence OR "antioxidant capacity" OR "oxidative damage" OR signal) AND (bird) AND (carotenoid OR carotenoids)

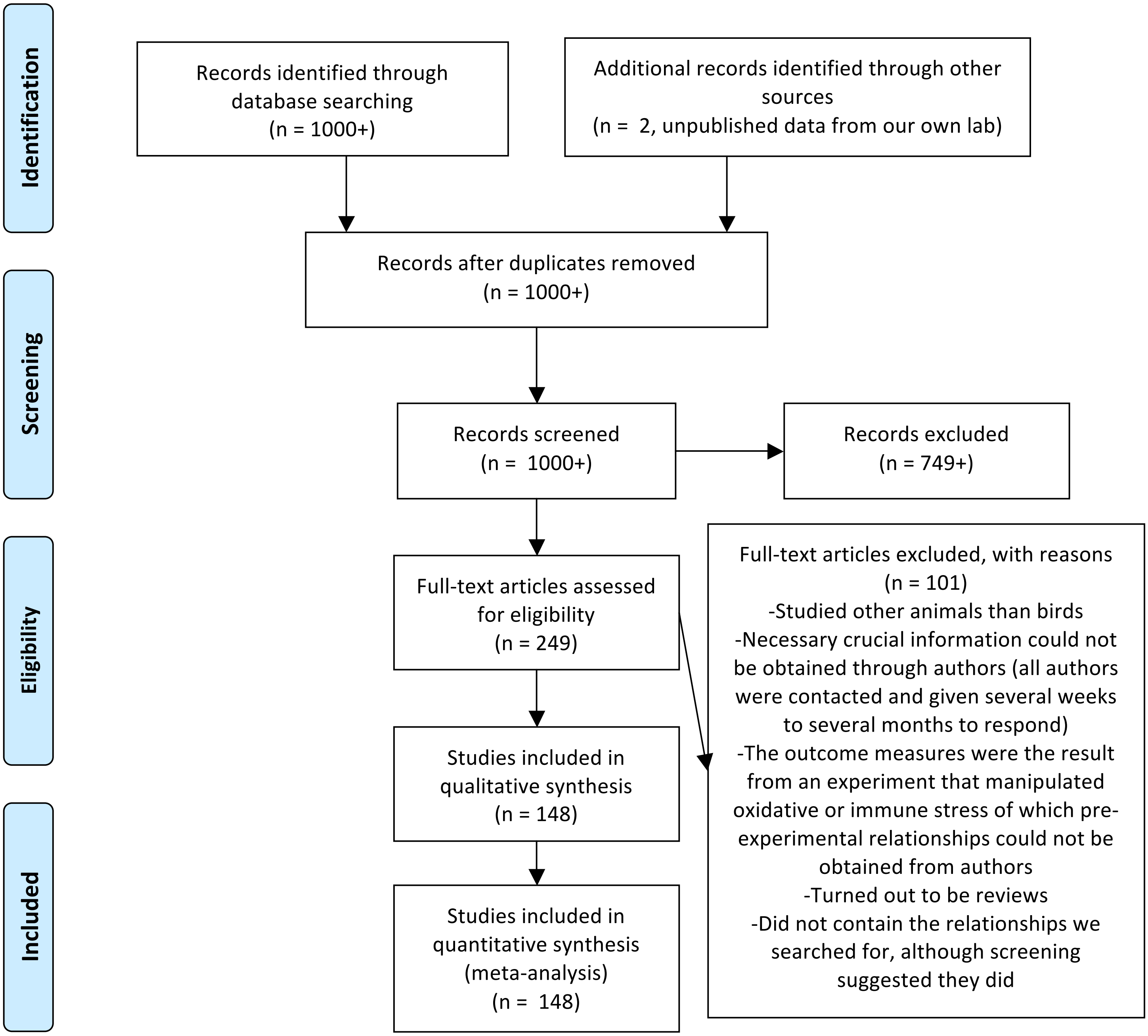

From: Moher D, Liberati A, Tetzlaff J, Altman DG, The PRISMA Group (2009). *P*referred *R*eporting *I*tems for *S*ystematic Reviews and *M*eta-*A*nalyses: The PRISMA Statement. PLoS Med 6(6): e1000097. doi:10.1371/journal.pmed1000097

For more information, visit www.prisma-statement.org.
