# Supplementary material for: What Does Carotenoid-Dependent Coloration Tell? Plasma Carotenoid Level Signals Immunocompetence and Oxidative Stress State in Birds–A Meta-Analysis
Source: PLoS One. 2012 Aug 14;7(8):e43088. doi: 10.1371/journal.pone.0043088 (PMC3419220; doi:10.1371/journal.pone.0043088)
Supplement: PRISMA S2 — PRISMA checklist for meta-analyses. (PDF) [file pone.0043088.s004.pdf]

PRISMA 2009 Checklist (corresponding to Simons et al. title: What does carotenoid-dependent coloration tell? Plasma carotenoid level signals immunocompetence and oxidative stress state in birds – a meta-analysis)

Section/topic	#	Checklist item	Reported in
**TITLE**			
Title	1	Identify the report as a systematic review, meta-analysis, or both.	title
**ABSTRACT**			
Structured summary	2	Provide a structured summary including, as applicable: background; objectives; data sources; study eligibility criteria, participants, and interventions; study appraisal and synthesis methods; results; limitations; conclusions and implications of key findings; systematic review registration number.	abstract
**INTRODUCTION**			
Rationale	3	Describe the rationale for the review in the context of what is already known.	Introduction
Objectives	4	Provide an explicit statement of questions being addressed with reference to participants, interventions, comparisons, outcomes, and study design (PICOS).	End of introduction
**METHODS**			
Protocol and registration	5	Indicate if a review protocol exists, if and where it can be accessed (e.g., Web address), and, if available, provide registration information including registration number.	Literature search
Eligibility criteria	6	Specify study characteristics (e.g., PICOS, length of follow-up) and report characteristics (e.g., years considered, language, publication status) used as criteria for eligibility, giving rationale.	Literature search
Information sources	7	Describe all information sources (e.g., databases with dates of coverage, contact with study authors to identify additional studies) in the search and date last searched.	Literature search
Search	8	Present full electronic search strategy for at least one database, including any limits used, such that it could be repeated.	In PRISMA flowchart and Literature search
Study selection	9	State the process for selecting studies (i.e., screening, eligibility, included in systematic review, and, if applicable, included in the meta-analysis).	Literature search
Data collection process	10	Describe method of data extraction from reports (e.g., piloted forms, independently, in duplicate) and any processes for obtaining and confirming data from investigators.	Literature search
Data items	11	List and define all variables for which data were sought (e.g., PICOS, funding sources) and any assumptions and simplifications made.	End of introduction and Figure 1, and within Color, Immune system components and

PRISMA 2009 Checklist (corresponding to Simons et al. title: What does carotenoid-dependent coloration tell? Plasma carotenoid level signals immunocompetence and oxidative stress state in birds – a meta-analysis)

			oxidative stress measures
Risk of bias in individual studies	12	Describe methods used for assessing risk of bias of individual studies (including specification of whether this was done at the study or outcome level), and how this information is to be used in any data synthesis.	Meta-analytic technique
Summary measures	13	State the principal summary measures (e.g., risk ratio, difference in means).	Meta-analytic technique
Synthesis of results	14	Describe the methods of handling data and combining results of studies, if done, including measures of consistency (e.g., I^2) for each meta-analysis.	Meta-analytic technique

Section/topic	#	Checklist item	Reported on page #
Risk of bias across studies	15	Specify any assessment of risk of bias that may affect the cumulative evidence (e.g., publication bias, selective reporting within studies).	Meta-analytic technique and funnel plots in supplement
Additional analyses	16	Describe methods of additional analyses (e.g., sensitivity or subgroup analyses, meta-regression), if done, indicating which were pre-specified.	Moderators
**RESULTS**			
Study selection	17	Give numbers of studies screened, assessed for eligibility, and included in the review, with reasons for exclusions at each stage, ideally with a flow diagram.	PRISMA flowchart supplement and Literature search
Study characteristics	18	For each study, present characteristics for which data were extracted (e.g., study size, PICOS, follow-up period) and provide the citations.	Moderators, supplement of datasets
Risk of bias within studies	19	Present data on risk of bias of each study and, if available, any outcome level assessment (see item 12).	This is not applicable
Results of individual studies	20	For all outcomes considered (benefits or harms), present, for each study: (a) simple summary data for each intervention group (b) effect estimates and confidence intervals, ideally with a forest plot.	supplement of datasets and funnel plots
Synthesis of results	21	Present results of each meta-analysis done, including confidence intervals and measures of consistency.	Figure 2
Risk of bias across studies	22	Present results of any assessment of risk of bias across studies (see Item 15).	Meta-analytic techniques and supplement of funnel plots

PRISMA 2009 Checklist (corresponding to Simons et al. title: What does carotenoid-dependent coloration tell? Plasma carotenoid level signals immunocompetence and oxidative stress state in birds – a meta-analysis)

Additional analysis	23	Give results of additional analyses, if done (e.g., sensitivity or subgroup analyses, meta-regression [see Item 16]).	Results section different subheadings
**DISCUSSION**			
Summary of evidence	24	Summarize the main findings including the strength of evidence for each main outcome; consider their relevance to key groups (e.g., healthcare providers, users, and policy makers).	Beginning of Discussion
Limitations	25	Discuss limitations at study and outcome level (e.g., risk of bias), and at review-level (e.g., incomplete retrieval of identified research, reporting bias).	Meta-analytic techniques
Conclusions	26	Provide a general interpretation of the results in the context of other evidence, and implications for future research.	End of discussion
**FUNDING**			
Funding	27	Describe sources of funding for the systematic review and other support (e.g., supply of data); role of funders for the systematic review.	Under funding

From: Moher D, Liberati A, Tetzlaff J, Altman DG, The PRISMA Group (2009). Preferred Reporting Items for Systematic Reviews and Meta-Analyses: The PRISMA Statement. PLoS Med 6(6): e1000097. doi:10.1371/journal.pmed1000097

For more information, visit: **www.prisma-statement.org**.
